# Pontocerebellar hypoplasia: a review from 1912 to 2022

**DOI:** 10.1093/braincomms/fcaf298

**Published:** 2025-08-17

**Authors:** Natalie A Kukulka, Shriya Singh, Matthew T Whitehead, William B Dobyns, Taeun Chang, Youssef A Kousa

**Affiliations:** Division of Neurology, Children’s National Hospital, Washington, DC 20010, USA; Division of Neurology, Children’s National Hospital, Washington, DC 20010, USA; Department of Radiology, Children’s National Hospital, Washington, DC 20010, USA; Division of Neuroradiology, Children’s Hospital of Philadelphia, Philadelphia, PA 19104, USA; Department of Radiology, Perelman School of Medicine, University of Pennsylvania, Philadelphia, PA 19104, USA; Department of Pediatrics, University of Minnesota, Minneapolis, MN 55455, USA; Division of Neurology, Children’s National Hospital, Washington, DC 20010, USA; Neonatal Neurology Program, Children’s National Hospital, Washington, DC 20010, USA; Division of Neurophysiology, Epilepsy, and Critical Care, Children’s National Hospital, Washington, DC 20010, USA; Department of Pediatrics, George Washington University School of Medicine and Health Sciences, Washington, DC 20052, USA; Department of Neurology, George Washington University School of Medicine and Health Sciences, Washington, DC 20052, USA; Division of Neurology, Children’s National Hospital, Washington, DC 20010, USA; Neonatal Neurology Program, Children’s National Hospital, Washington, DC 20010, USA; Division of Neurophysiology, Epilepsy, and Critical Care, Children’s National Hospital, Washington, DC 20010, USA; Department of Pediatrics, George Washington University School of Medicine and Health Sciences, Washington, DC 20052, USA; Department of Neurology, George Washington University School of Medicine and Health Sciences, Washington, DC 20052, USA; Department of Genomics and Precision Medicine, George Washington University School of Medicine and Health Sciences, Washington, DC 20052, USA

**Keywords:** pontocerebellar hypoplasia, posterior fossa malformation, prenatal brain development, neurodevelopmental disorders, neurodegenerative diseases

## Abstract

Pontocerebellar hypoplasia is a rare neurodevelopmental disorder that results from differences in formation and function of the pons, cerebellum and cerebrum. It can be diagnosed prenatally or postnatally with a combination of clinical, neuroimaging and genetic data obtained over time. The diagnosis of pontocerebellar hypoplasia usually portends severe developmental delay, epilepsy and/or neurodegeneration in childhood. Here we perform a comprehensive review with the primary goal of evaluating published evidence addressing the clinical and genetic features of pontocerebellar hypoplasia by type and subtype. Secondly, we summarize neurodiagnostic patterns of pontocerebellar hypoplasia and demonstrate its spectrum. Finally, we provide recommendations in diagnosis, prognosis and management for the neurologist. To address these goals, we performed an extensive review of published literature from 1912 to 2022. We identified 191 publications by combining search results from PubMed, OMIM and cross-referenced bibliographies. Publications on developmental neuroanatomy, not pertaining to pontocerebellar hypoplasia or published in a foreign language were excluded. We performed both qualitative (1912–1993) and quantitative (1993–2022) analyses to understand the current classification of this disease as it pertains to genetic and neurodiagnostic features of pontocerebellar hypoplasia by type and subtype. Our review shows that the most reported types of pontocerebellar hypoplasia are 1, 2 and 6; less frequently described are 3, 4 and 9. Very few cases are described for all other subsequent pontocerebellar hypoplasia types. Mutations in *TSEN54*, *RARS2*, *EXOSC3* and *AMPD2* (genes that regulate RNA processing and basic cellular metabolism) are the most frequently reported pathological mutations in pontocerebellar hypoplasia. The neuroradiographic features of pontocerebellar hypoplasia are complex and evolve over time, affecting the pons, cerebellum, vermis, cortex and cerebral white matter. In conclusion, pontocerebellar hypoplasia is a rare neurodevelopmental disorder, often the result of genetic dysfunction in basic neural metabolism. The diagnosis conveys significant implications for the affected individual and their families and requires a combination of clinical, neuroradiographic, and genetic testing to best inform type/subtype categorization of pontocerebellar hypoplasia.

## Teaser

Kukulka *et al*. report that pontocerebellar hypoplasia is most often linked to mutations in *TSEN54*, *RARS2*, *EXOSC3* and *AMPD2*, with Types 1, 2, and 6 being most common. They propose a dyadic gene-based classification to improve diagnostic clarity, guide clinical management and highlight research opportunities.

## Graphical Abstract

**Graphical Abstract:**
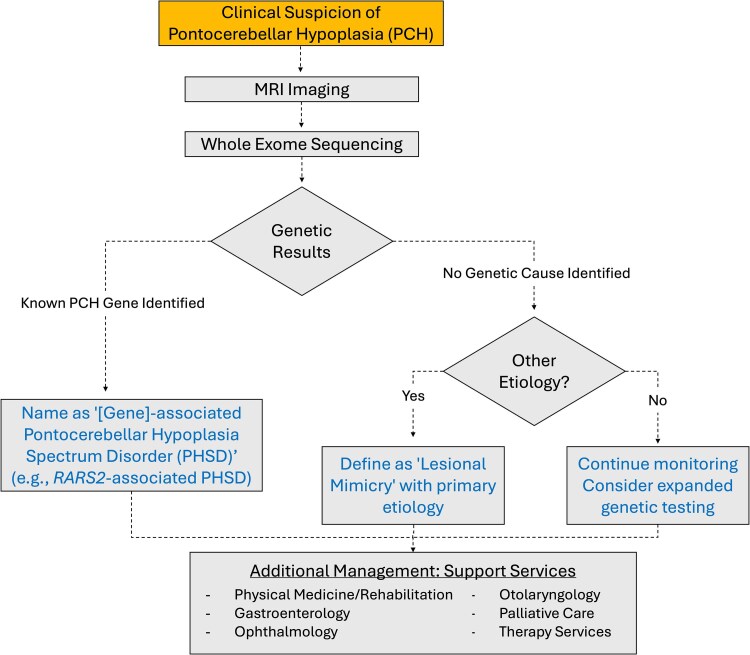

## Introduction

Pontocerebellar hypoplasia (PCH) is a clinical disorder that results from abnormal growth or maturation of the pons and cerebellum due to differences in neurodevelopmental programming. Concurrently, it can also indicate a relative lack of age-appropriate volumetric growth of the pons and cerebellum without known clinical implications. Neuroanatomical disorders of the pons and cerebellum likely result from chromosomal/genetic abnormalities affecting neural migration or metabolic derangements in the metencephalon at or after the sixth week of gestation. In the interest of lexicon clarity and consistency, there is a movement in the field to reserve the term ‘PCH’ for disorders with suspected or confirmed specific genetic mutations carrying a clinically significant phenotype. While the aetiology of PCH is rooted in numerous genetic mutations, the contribution of exogenous, endogenous, and behavioural factors constituting the neural exposome remains poorly understood, necessitating further investigation.^1^

Recent advances in genetic sequencing have facilitated growing sub-categorization in diagnosing PCH as a disorder with sometimes variable clinical significance. Current literature has expanded to 16 PCH types, with additional A–F subtypes for PCH 1 and 2, yielding 26 different disease categories. With few exceptions, we now recognize that sequencing data are not always clinically predictive because mutations in the same gene can lead to different PCH subtypes (allelic heterogeneity) and mutations in different genes can lead to the same PCH subtype (locus heterogeneity).^2-6^ In parallel, advances in neuroimaging and quantitative volumetric analyses have provided greater resolution of posterior fossa structures, further increasing diagnostic suspicion for more PCH subtypes. The heterogeneity and rarity of PCH have limited our ability to make substantive progress in the field towards disease-alleviating therapies. As a result, there is increasing difficulty in identifying, diagnosing and managing patients and counselling their families.

To bridge the gap and advance the field further, we performed the most comprehensive qualitative and quantitative literature review of PCH to date. We defined PCH as a constellation of specific radiographic, clinical and genetic features, currently recognized by Online Mendelian Inheritance in Man® (OMIM) and not explained by another disease aetiology. We did not include otherwise known genetic syndromes with an associated radiographic feature of hypoplastic pons and cerebellum. This review aims to summarize over 100 years of PCH literature (from 1912 to 2022), offer a comprehensive overview of the historical and current categorization of PCH as a disease, and provide the most up-to-date phenotypic, genetic and neuroimaging characteristics of the disease by type and subtype. In addition, this work highlights research opportunities and serves as an educational resource not only neonatal neurologists but for the broader neurology community—especially in light of the recent United Council for Neurologic Subspecialties approval of a neonatal neurocritical care neurology fellowship.

## Methods

**Figure 1:**
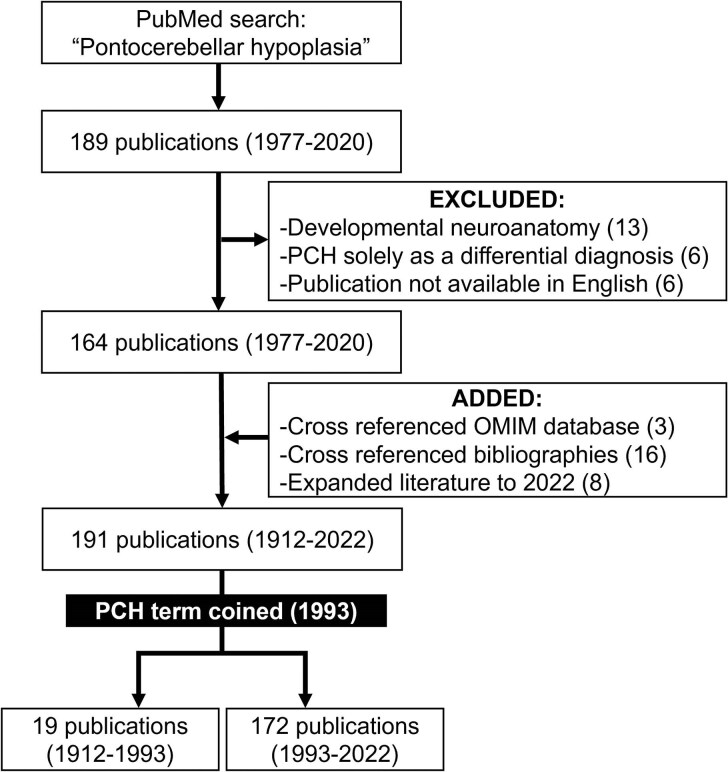
**Literature selection.** A PubMed search for the term ‘pontocerebellar hypoplasia’ identified 189 publications. Excluded manuscripts included those addressing (i) developmental neuroanatomy of the pons and cerebellum, (ii) clinical cases identifying PCH only as differential diagnosis and (iii) publications not written in English. Using these criteria, we identified 164 publications between 1977 and 2020. Cross-referencing OMIM, available bibliographies and extending the publications to 2022 yielded a total of 191 manuscripts (1912–2022). The term PCH was coined in 1993. As a result, we divided the literature into a set of publications for qualitative (1912–1993) and quantitative analyses (1993–2022). OMIM, Online Mendelian Inheritance in Man®; PCH, pontocerebellar hypoplasia.

To conduct this narrative review, we began by searching PubMed using the term ‘pontocerebellar hypoplasia’ and did not exclude any search terms (Fig. 1), which yielded 189 relevant publications. Reviewing each of these, we excluded publications addressing only developmental neuroanatomy (*n* = 13), text not written in English (*n* = 6) or when the diagnosis of PCH was only discussed as a differential diagnosis (*n* = 6). Each manuscript’s bibliography was then reviewed and cross-referenced with OMIM, yielding 19 additional publications. We subsequently expanded the timeline to include manuscripts until 2022, thus adding eight more relevant publications. In total, we identified and comprehensively reviewed 191 publications between 1912 and 2022 for this review. To avoid misclassification bias, review and classification of the literature was performed by one individual, and then critically reviewed and evaluated by another individual. We extracted information on reported cases, genetic aetiologies and neuroradiographic features by PCH type and subtype.

The term PCH was coined by Barth *et al*. in 1993, which corresponded with the beginning of notable advances in neuroimaging and genetic sequencing.^7,8^ We qualitatively analysed 19 manuscripts prior to 1993 and proposed a current PCH categorization (Table 1). Manuscripts, including and after 1993, were incorporated in the quantitative analyses. When available, information on the observed inheritance pattern and availability of translational research systems was obtained. Publications addressing more than one form of PCH were included with each type addressed.

**Table 1:** Historical review of the PCH literature

Authors	Year	Significant events	Initial classification	Proposed classification
Vogt and Astwazaturow^9^	1912	1st neuropathological account of PCH	Combination of neocerebellar and pontine hypoplasia	Unavailable
Brun^10^	1917	Combination of hypoplasia + neuronal atrophy + choreiform movements	Neocerebellar hypoplasia	PCH 2
Brouwer^11^	1924	1st neuropathological report in a patient **Attempt to name the disorder**	**Hypoplasia ponto-neocerebellaris**	PCH 2
Koster^12^	1926	Further neuropathological evaluation	**Hypoplasia ponto-neocerebellaris**	PCH 2
Biemond^13^	1955	Dentate malformation must be considered the core and cause of the ponto-neocerebellar hypoplasia	Ponto-neocerebellar hypoplasia	PCH 1
Norman and Urich^14^	1958	Validation of Brouwer’s principle of systemic degeneration; new link between malformation and abiotrophy	Cerebellar hypoplasia associated with systemic degeneration in early life	PCH 4
Norman^15^	1961	1st description of PCH + anterior horn disease	Cerebellar hypoplasia in Werdnig–Hoffmann disease	PCH 1
Weinberg and Kirkpatrick^16^	1975	Supportive case for Norman and Urich	Cerebellar hypoplasia in Werdnig–Hoffmann Disease	PCH 1
Goutières, Aicardi, and Farkas^17^	1977	Authors proposed the disease is distinct from Werdnig–Hoffmann disease	Anterior horn cell disease associated with PCH in infants	PCH 1
Peiffer and Peiffer^18^	1977	1st description of PCH + microcephaly + chorea	Dysplastic neocortical architecture	PCH 2
Steiman *et al*.^19^	1980	Mild PCH + anterior horn disease **Attempt to name the disorder**	**Infantile neuronal degeneration**	PCH 1
Herrick *et al*.^20^	1983	Support for progressive multisystem atrophy; thought to represent autosomal recessive inheritance	Could represent ponto-neocerebellar hypoplasia or olivo-ponto-cerebellar atrophy	PCH 4
de León *et al*.^21^	1984	**Attempt to name the disorder**	**Amyotrophic cerebellar hypoplasia**	PCH 1
Kawagoe and Jacob^22^	1986	Disease as a result of inactivity-abiotrophia caused by the hypoplastic and insufficiently functional neocerebellar cortex	Neocerebellar hypoplasia with systemic combined olivo-ponto dentatal degeneration	PCH 4
De Caro *et al*.^23^	1987	Histological determination of the pathology and chronology of lesions	Bulbopontocerebellar hypoplasia with aplasia of the inferior olivary nucleus	PCH 4
Barth *et al*.^2^	1990	Study confirmed the disease as an inherited neurodegenerative disease	Systemic atrophy with early onset	PCH 2
Kamoshita *et al*.^24^	1990	**Attempt to name the disorder**	**Norman syndrome**	PCH 1
Barth^25^	1992	Review of previously published literature plus case report of siblings with PCH	Pontocerebellar hypoplasias	PCH 1
Albrecht *et al*.^3^	1993	Report of previously not known familial infantile encephalopathy with olivopontocerebellar hypoplasia	Fatal infantile encephalopathy with olivopontocerebellar hypoplasia and microencephaly	PCH 4
Barth^7^	1993	Proposed delineation among PCHs **Attempt to name the disorder**	**PCH Type-1 and PCH Type-2**	PCH 1 and PCH 2

The first manuscript describing PCH was published in 1912, and since then several publications have described the clinical entity using different nomenclature. Using available information, we attempt to classify each of these based on current disease classification. Most cases were PCH Types 1, 2 and 4.

To craft a comprehensive review, we meticulously categorized publications by the significance of their contribution to the field. Categories included standalone case report/series and those with histopathological, biochemical, prenatal, genetic or radiographic data. This was intended to address the multidisciplinary approach to the disease that included contributions from laboratory researchers, pathologists, neurologists, geneticists and radiologists. Reviews, commentaries, basic research, morphometric and natural history studies were also evaluated. The literature was then chronologically evaluated and sorted by PCH type, subtype and genetic aetiology. Focusing on neuroimaging data, we analysed the range and commonality of radiographic characteristics by disease type and subtype and created a comprehensive table summarizing such findings. To demonstrate the intricacy of PCH diagnosis as a disorder, we provide a radiographic example of cerebellar hypoplasia as an isolated imaging finding and as PCH 6 over time. This study was completed from 2019 to 2023 under IRB approval that qualified with research exemption (protocol number 12020) at Children’s National Hospital.

## Results

### Literature review

The first description of a hypoplastic pons and cerebellum was in 1912 by Vogt and Astwazaturow (historical perspective since 1912 reviewed in Table 1 and Supplementary Fig. 1).^9^ Applying current classification, cases published prior to 1993 could be consistent with the diagnosis of PCH Types 1, 2 and 4, yet classification was challenging due to changes in nomenclature (Table 1).^2,3,26-32^ Since 1993, research on PCH further evolved with a notable increase in publications including genetic sequencing starting in 2011, which continued through 2022 (Supplementary Fig. 2). As demonstrated by Supplementary Fig. 2, organized in 5-year brackets, we see a trend for more research publications with novel model systems (3 publications in 2008–2012 increased to 12 in 2018–2022). It is important to highlight that there has only been one natural history study on PCH 2A, published in 2014,^33^ and only two studies with sole focus on prenatal investigation of PCH 2, published in 2010.^34,35^ Over the last century, there are only two studies with extensive biochemical analyses of PCH: one investigated the enzyme activities of the respiratory chain in PCH6 and OXPHOS complex,^36^ and the other evaluated mitochondrial dysfunction with recessive mutations in *EXOSC3* with PCH. Publications discussing radiographic patterns have been rare with a total of six case reports focusing on MRI data and three morphometric studies (1992–2022). While the number of PCH publications grew in 2008, so did the classification of PCH. This growth required a more detailed examination of individual types, subtypes and genetic causes.

### Inconsistencies in PCH categorization

We found discrepancies in categorizing disorders with the same genetic aetiology. Often, different names were used. For example, progressive cerebello-cerebral atrophy (PCCA) Type 1 and PCH 2D are both due to *SEPSECS* mutations, and PCCA Type 2 and PCH 2E are due to *VPS53* mutations.^37^ Some have reported new mutations under PCH subtypes that are still not widely recognized or included in OMIM, such as *PLA2G6* as a PCH 1 subtype^38^ or *CASK* mutations as part of PCH 3.^39^ Other notable discrepancies are between the literature and OMIM. Although the *TSEN* c.919G>T mutation is listed for PCH Types 1, 2, 4 and 5, the connection between *TSEN* mutation and PCH 1 is not published in OMIM.^40^ In addition, in 2017, van Dijk *et al*. classified the *SLC24A46* mutation as PCH 1D;^41^ however, subsequent literature and OMIM refer to it as PCH 1E.

### Clinical features and associations with PCH

As reported, PCH is clinically heterogeneous. In fact, there is no single clinical feature shared in the diagnosis of PCH, neither among the 26 subtypes nor within the 16-type classification. However, a few unifying features are identified. First, many PCH types have an onset early in life, from prenatal to infancy. Second, many have developmental delays and/or abnormal muscle tone (hypertonia or hypotonia). Third, many affected patients develop epilepsy, which might be refractory to treatment. To provide a comprehensive review of current categorization of PCH, we incorporated previously recognized clinical features of each disorder (Table 2; see Supplementary Table 1 for the complete table). As displayed in the table, the diagnosis of PCH by subtype is made by clinically and/or radiologically distinguishing features. For instance, PCH 1 has been associated with polyneuropathy and contractures, while PCH 2 is recognized by extrapyramidal dyskinesias and abnormal movements (of eyes or limbs). Similarly, PCH 4 is distinguished by fatal/neonatal apnoeas, whereas there is a serum/CSF lactic acidosis in PCH 6. Due to the XY reversal in PCH Type 7, affected infants can have ambiguous genitalia.^50^ Furthermore, not all types have been known to be neurodegenerative in nature, including PCH 8 and PCH 11.

**Table 2:** Summary of PCH Categorization

PCH phenotype	Locus	IHT	Gene	Gene function	First report in PCH	Gene identification in PCH	Clinical features	PCH animal models
PCH Type 1								
Type 1A	14q32.2	AR	*VRK1*	Neuronal migration	Norman (1961)^42^	Renbaum *et al*. (2009)^15^	Onset prenatal to late infancy, SMA phenotype, polyneuropathy	Mouse: Vinograd-Byk *et al*. (2015)^43^
Type 1B	9p13.2	AR	*EXOSC3*	mRNA degradation	Ryan *et al*. (2000)^26^	Wan *et al*. (2012)^44^	Neonatal onset, early neonatal apnoea, oculomotor apraxia (+/−), optic atrophy (+/−), tongue fasciculations, contractures, axonal motor neuropathy	None
Type 1C	13q13.3	AR	*EXOSC8*	mRNA degradation	Boczonadi *et al*. (2014)^28^	Boczonadi *et al*. (2014)^28^	Onset in first months of life, respiratory failure, contractures, SMA phenotype, atrophy, hearing impairment	Zebrafish: Boczonadi *et al*. (2014)^28^
Type 1D	4q27	AR	*EXOSC9*	mRNA degradation	Burns *et al*. (2018)^31^	Burns *et al*. (2018)^31^	Onset at birth to early infancy, reduced foetal movements, early neonatal apnoea, contractures, fasciculations, impaired pursuit, axonal motor neuronopathy	Zebrafish: Burns *et al*. (2018)^31^
Type 1E	5q22.1	AR	*SLC25A46*	Mitochondrial fission/fusion	Wan *et al*. (2016)^30^	Wan *et al*. (2016)^30^	Prenatal onset, neonatal lethal, polyhydramnios, early neonatal apnoea, contractures, optic atrophy (+/−), polyneuropathy	Zebrafish: Wan *et al*. (2016)^30^
Type 1F **#**	10q24.1	AR	*EXOSC1*	mRNA degradation	Somashekar *et al*. (2021)^32^	Somashekar *et al*. (2021)^32^	Onset in infancy, developmental delays, blue sclera, microcephaly, dysmorphic facies, hypotonia, diminished reflexes	None
PCH Type 2								
Type 2A	17q25.1	AR	*TSEN54*	tRNA splicing	Barth *et al*. (1990)^2^	Budde *et al*. (2008)^4^	Onset at birth, impaired swallowing, central visual impairment, hypertonia at birth, extrapyramidal dyskinesia	None
Type 2B	3p25.2	AR	*TSEN2*	tRNA splicing	Barth *et al*. (1990)^2^	Namavar *et al*. (2011)^6^	Onset at birth, central visual impairment, hyperkinetic involuntary movements	None
Type 2C **#**	19q13.42	AR	*TSEN34*	tRNA splicing	Barth *et al*. (1990)^2^	Budde *et al*. (2008)^4^	Onset at birth, extrapyramidal dyskinesia	None
Type 2D	4p15.2	AR	*SEPSECS*	Selenocysteine synthesis catalyst	Ben-Zeev *et al*. (2003)^27^	Agamy *et al*. (2010)^45^	Onset in infancy, contractures, sleep disturbances, irritability, oedema of face and limbs, polyneuropathy, optic atrophy (+/−)	None
Type 2E	17p13.3	AR	*VPS53*	Retrograde transport of endosomes to Golgi	Feinstein *et al*. (2014)^29^	Feinstein *et al*. (2014)^29^	Onset in infancy, progressive, failure to thrive, gaze-evoked nystagmus (+/−), optic atrophy (+/−), distal limb oedema (+/−)	None
Type 2F	1q25.3	AR	*TSEN15*	tRNA splicing	Barth *et al*. (1990)^2^	Breuss *et al*. (2016)^46^	Onset at birth, poor or absent fixation	None
PCH Type 3								
Type 3 **#**	7q21.11	AR	*PCLO*	Regulation of synaptic protein/vesicle formation	Rajab *et al*. (2003)^47^	Ahmed *et al*. (2015)^48^	Onset at birth, neonatal hypotonia, optic atrophy	Rat: Falck *et al*. (2020)^49^
PCH Type 4								
Type 4	17q25.1	AR	*TSEN54*	tRNA splicing	Albrecht *et al*. (1993)^3^	Budde *et al*. (2008)^4^	Prenatal onset, death in infancy, polyhydramnios, early neonatal apnoea, contractures	None
PCH Type 5								
Type 5 **#**	17q25.1	AR	*TSEN54*	tRNA splicing	Patel *et al*. (2006)^5^	Namavar *et al*. (2011)^6^	Prenatal onset, death in neonatal period, seizure including seizure-like activity *in utero* starting around 18 weeks gestation, polyhydramnios, early neonatal apnoea	None

IHT, inheritance; AR, autosomal recessive; SMA, spinal muscular atrophy .

A subset of the PCH type/subtypes (1A–16) are listed with its locus, associated genetic aetiology, protein function, inheritance pattern, OMIM IDs, publications first reporting the disease and associated genetic aetiology, and availability of translational/model research systems. A hashtag (**#**) indicates a provisional relationship between the phenotype and gene per OMIM. A complete version of this table, including the full list of PCH type/subtypes is provided in Supplementary Table 1.

Of importance to management are also reports of unique clinical associations seen in patients with a PCH diagnosis. For instance, PCH 2 was reported in a case of recurrent rhabdomyolysis.^51^ PCH 3 was reported in conjunction with severe Vitamin A deficiency^52^ and separately in a case of tetralogy of Fallot.^53^ Infantile spasms or early myoclonic encephalopathy were identified in a patient with PCH 6,^54^ and Stickler Syndrome Type 2 was identified in patient with PCH 9.^55^ Radiographically, PCH 1B was reported with rhombencephalosynapsis and microlissencephaly.^56^ In an effort to avoid anchoring bias, recognizing these features and associations can be useful in diagnosing, managing and treating affected patients.

### Most frequently reported PCH type/subtype and associated genetic aetiologies

**Figure 2:**
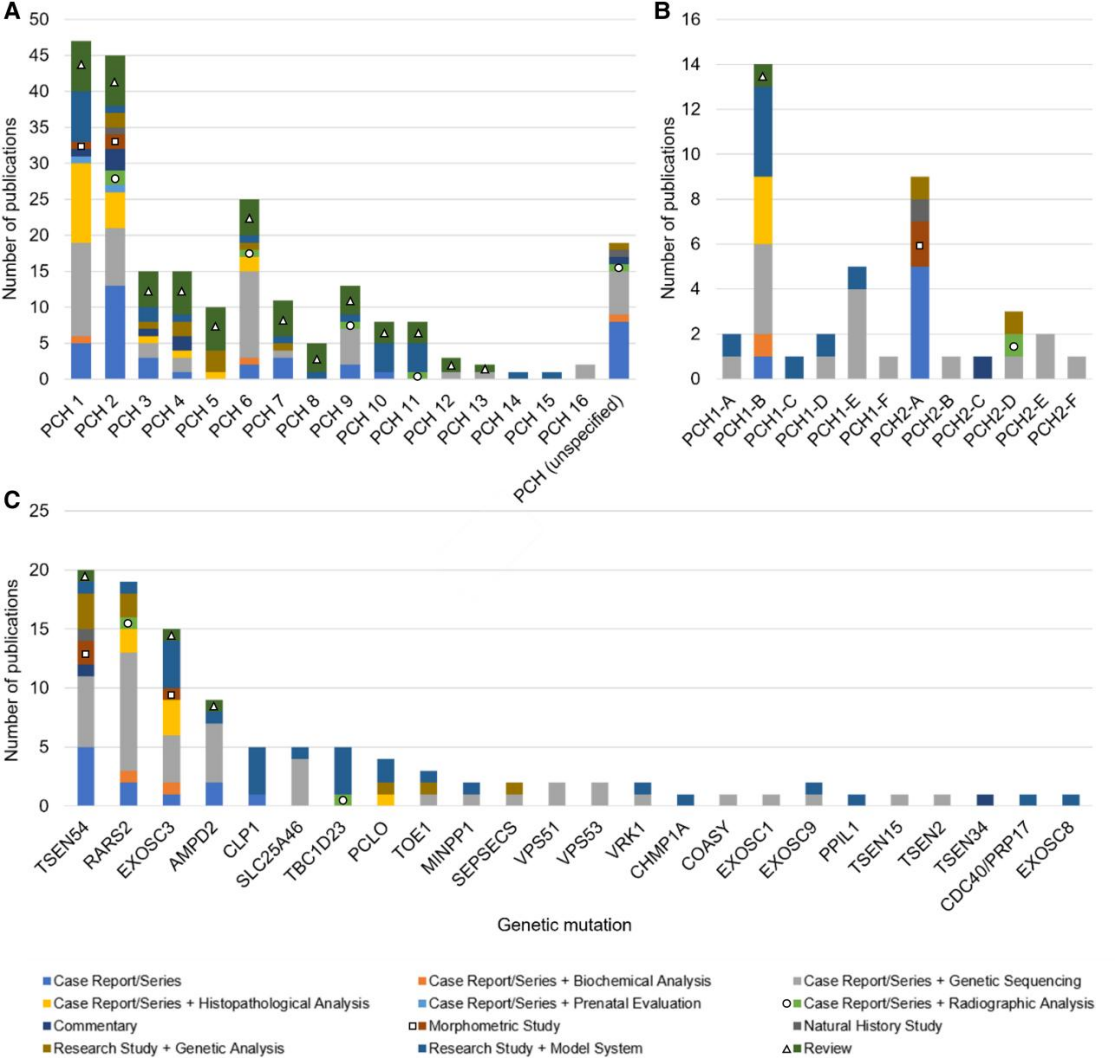
**Studies performed on PCH by type/subtype and reported genetic aetiologies.** The figure represents summed values of publications on PCH (*y*-axis) sorted by (**A**) PCH type (*x*-axis), (**B**) PCH subtype (*x*-axis) and (**C**) genetic aetiology. Staggered columns are colour-coded by the type of study published. The most frequently reported PCH types are 1, 2 and 6. Less frequently reported are 3, 4 and 9. The most frequently reported subtypes are 1-B, 2-A and 1-E. There are 24 associated genetic aetiologies, and the most reported mutations include the *TSEN54*, *RARS2*, *EXOSC3* and *AMPD2* genes. To support broader accessibility, we have incorporated shape-based annotations: ■ (square for red), ● (circle for light green), and ▴ (triangle for dark green).

PCH 1 (*n* = 47), 2 (*n* = 45) and 6 (*n* = 25) and Subtypes 1-B (*n* = 14), 2-A (*n* = 9) and 1-E (*n* = 5) are among the most frequently reported (Fig. 2A and B). Types 12–16 are described in less than a total of five publications over the past 29 years, starting with manuscripts after 2018, with emphasis on basic research or case report/series with contributory novel sequencing. As with other diseases, mutations in the same gene can lead to different types of PCH (allelic heterogeneity) and mutations in different genes can lead to the same PCH type (locus heterogeneity).^2-6^ The most frequently reported genetic aetiology among 24 candidate genes reported include *TSEN54* (*n* = 20), *RARS2* (*n* = 19), *EXOSC3* (*n* = 15) and *AMPD2* (*n* = 9) (Fig. 2C). The 20 remaining genes are each reported in five citations or less (Fig. 2C). Reported gene function often involves RNA synthesis or processing (see Gene function column, Table 2). Chromosome 17 includes most PCH loci (2A, 2E, 4, 5 and 12, which result from mutations in *TSEN54*, *COASY* and *VPS53*) (Table 3). To complement genetic and clinical findings, preclinical models, including yeast, zebrafish, mice and rats, have been developed for several PCH types and subtypes, providing early insights into disease mechanisms (see PCH animal models column, Table 2).

**Table 3:** Breakdown of PCH subtypes by locus and gene

Locus	PCH Subtype	Gene
Chromosome 1		
1p13.3	PCH Type 9	*AMPD2*
1q25.3	PCH Type 2F	*TSEN15*
1p34.1	PCH Type 7	*TOE1*
Chromosome 3		
3q12.1-q12.2	PCH Type 11	*TBC1D23*
3p25.2	PCH Type 2B	*TSEN2*
Chromosome 4		
4p15.2	PCH Type 2D	*SEPSECS*
4q27	PCH Type 1D	*EXOSC9*
Chromosome 5		
5q22.1	PCH Type 1E	*SLC25A46*
Chromosome 6		
6q15	PCH Type 6	*RARS2*
6p21.2	PCH Type 14	*PPIL1*
6q21	PCH Type 15 #	*CDC40*
Chromosome 7		
7q21.11	PCH Type 3 #	*PCLO*
Chromosome 9		
9p13.2	PCH Type 1B	*EXOSC3*
Chromosome 10		
10q23.2	PCH Type 16	*MINPP1*
10q24.1	PCH Type 1F #	*EXOSC1*
Chromosome 11		
11q12.1	PCH Type 10	*CLP1*
11q13.1	PCH Type 13	*VPS51*
Chromosome 13		
13q13.3	PCH Type 1C	*EXOSC8*
Chromosome 14		
14q32.2	PCH Type 1A	*VRK1*
Chromosome 16		
16q24.3	PCH Type 8	*CHMP1A*
Chromosome 17		
17p13.3	PCH Type 2E	*VPS53*
17q21.2	PCH Type 12	*COASY*
17q25.1	PCH Type 2A	*TSEN54*
17q25.1	PCH Type 4	*TSEN54*
17q25.1	PCH Type 5 **#**	*TSEN54*
Chromosome 19		
19q13.42	PCH Type 2C **#**	*TSEN34*

Ordered by chromosome number and locus, this table highlights that many PCH types/subtypes are linked to single loci. A hashtag (**#**) indicates a provisional relationship between the phenotype and gene per OMIM.

### Analysis of the neuroimaging literature

We found that underdevelopment of the pons and cerebellum is usually, but ‘not invariably’, present to some degree in newborns. Reductions in volume may not be present, qualitatively or quantitatively, in the early and mid-prenatal period.^6,57-59^ As a result, it is difficult to exclude the diagnosis of PCH in the prenatal and neonatal stages, and follow-up imaging is often needed, especially when the diagnosis is postulated with clinical course or sequencing data. Summarized below are key imaging considerations.

### Utility of prenatal ultrasound in diagnosing PCH

Prenatal ultrasound is widely accessible, costs less than MRI and is routinely used in clinical practice, often providing first views of the developing brain. However, ultrasound may be unreliable and insufficiently sensitive for the diagnosis, especially at earlier gestational ages. In most cases, prenatal ultrasounds were either normal or revealed an enlarged cisterna magna in isolation or hypoplastic posterior fossa structures with or without cerebral abnormalities.^6,60^ There were exceptions in the literature (e.g. foetal ultrasound performed at 20 and 28 weeks’ gestation revealed PCH and microcephaly in foetuses with *COASY* gene defects corresponding to PCH 12).^5,61,62^ However, in a series of five cases imaged between 22 and 25 weeks and confirmed postnatally, ultrasound did not show diagnostic features of PCH.^58^ Among these cases, cerebellar hypoplasia was first identified only at 30 weeks in a single patient. Similarly, in a twin gestation with documented *TSEN54* mutations at 20 weeks, cerebellar hypoplasia was first detected at 31 weeks.^34^ As a result, diagnosing PCH sonographically may not be feasible until later in the third trimester. Thus, follow-up evaluation should be considered, especially when there is an accompanying suspicion for a neurogenetic disorder.

### Prenatal MRI

Compared with ultrasound, prenatal MRI is most useful in evaluating structural details of the posterior fossa parenchyma and its coverings.^63^ It complements and often enhances sonographic assessments with its superior signal-to-noise ratio. Furthermore, prenatal MRI allows for a more complete and uniform view of the entire brain. While volumetric measurements (3D) may be superior to 2D measurements, they require thinner section acquisition (generally <2 × 2 × 2 mm voxel size) that extends the duration of the scan and, consequently, increases the risk of foetal motion artefact.^64-66^ Compared to conventional 1.5T MRI, the 3T prenatal MRI usually offers better anatomic detail of the cerebellum and brainstem, but at the expense of prolonging scan time to offset the increased specific absorption rate.^67,68^

There is scarce literature regarding diagnosis and assessment of PCH using prenatal brain MRI. However, in some instances, prenatal MRI can be diagnostic. For example, PCH, corpus callosum hypoplasia and cerebral white matter hypoplasia associated with *AMPD2*-related PCH 9 was diagnosed at 24 and 30 weeks’ gestation.^69^ The cardinal feature of PCH 9, a midbrain with ‘figure of 8’ appearance, was identified at 30 weeks.^69^

### Post-natal MRI

**Figure 3:**
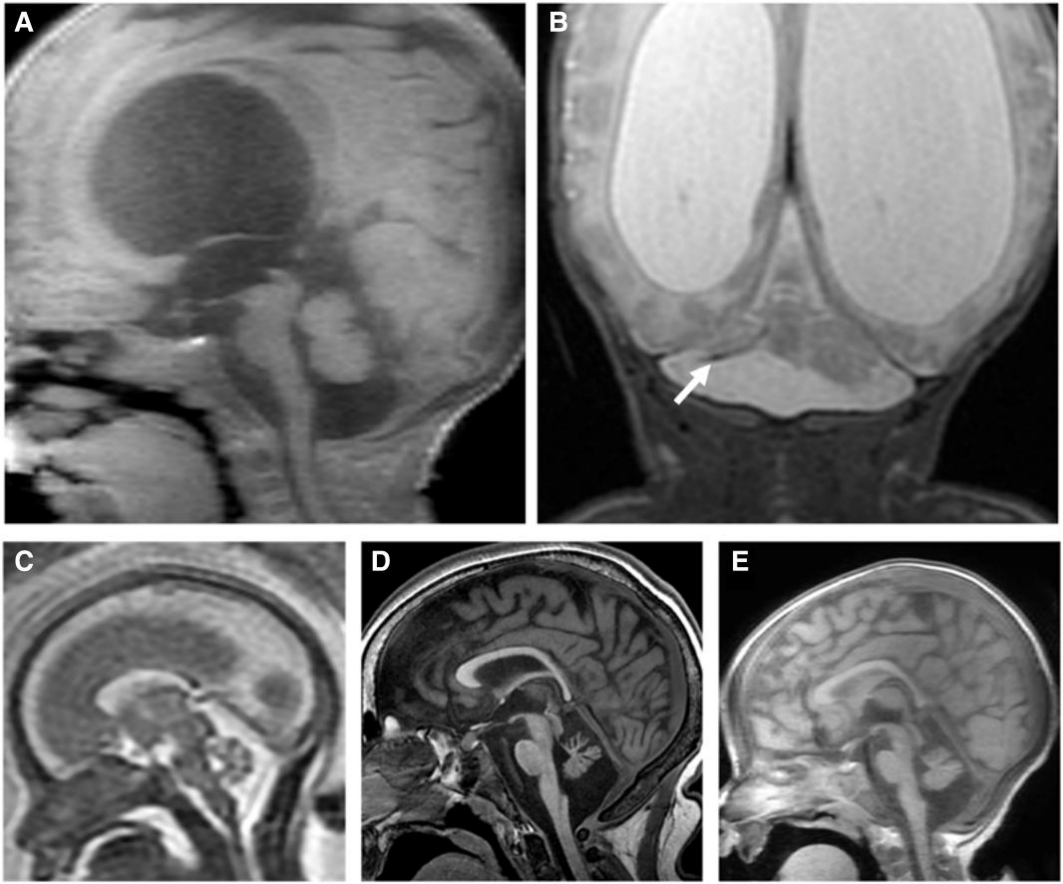
**Neuroimaging findings of PCH.** (**A**) Sagittal T1WI (TR/TE ms, 12/5) and (**B**) coronal T2WI (TR/TE ms, 2502/44) from a 3-month-old, former 27-week premature infant, with resulting cerebellar injury, show the ‘dragonfly’ sign of cerebellar hypoplasia. Note visible volume loss, more pronounced in the cerebellar hemispheres than the vermis. Post-haemorrhagic hydrocephalus is also present along with hemosiderin staining from prior haemorrhage seen along the under-surface of the right cerebellar hemisphere (arrow, **B**). Images (**C–E**) demonstrate radiographic findings in a patient with a *RARS2* mutation (PCH6). Sagittal T2WI (TR/TE ms, 5000/161) (**C**) from a foetal MR at 22 weeks gestation shows the cerebrum, cerebellum and brainstem to be of age-appropriate size and morphology. Post-natal sagittal T1WI MR exams (TR/TE ms, 8–11/3–5) at 7 months (**D**) and 10 years (**E**) reveal development of PCH with progressive cerebellar atrophy and lack of appropriate pontine growth. Images were obtained from patients seen at CNH under an IRB-approved protocol.

Post-natal non-sedate brain MRI is useful in identifying, following-up and confirming a suspected diagnosis of PCH, even when the genetic aetiology is uncertain. In the neonatal period, this can be done ‘feed-and-bundle’ style without anaesthesia. A standard brain MR protocol at Children’s National Hospital includes volumetric T1-weighted imaging (T1WI), axial T2, axial fluid-attenuated inversion recovery (FLAIR) (>1 year) or proton density (<1 year), axial diffusion tensor imaging, axial 3D gradient echo and coronal fat sat T2-weighted imaging (T2WI). A diffusion-weighted sequence is useful in excluding ischaemic or infectious PCH mimics (Fig. 3) and in considering *RARS2*-related aetiologies (Fig. 3). In such cases, loss of *RARS2* leads to insufficient mitochondrial tRNS-arg charging and a phenotype similar to patients who have a genetic difference affecting the mitochondrial respiratory chain. Thus, diffusion-weighted imaging (DWI) is useful in detecting neurometabolic decompensation for at least some PCH cases.^70^ Diffusion tensor imaging is an advanced MR technique that can quantify the degree and extent of white matter fibre deficiency and is potentially useful in distinguishing PCH from non-progressive hypoplasia and progressive atrophy.^71^

### Pathognomonic imaging features of PCH

There can be distinguishing features among PCH types and these warrant specific mention when the diagnosis is suspected (Table 4). For example, cerebellar hypoplasia without pontine hypoplasia is often found in PCH 1.^59,72,73^ Anterior horn involvement in PCH 1 can theoretically lead to reduced spinal cord volume in mimicking spinocerebellar ataxia; however, we did not find such examples by MRI. *TSEN54* mutations are strongly associated with the appearance of a ‘dragonfly’ cerebellum, showing disproportionate hypoplasia of the hemispheres (dragonfly wings) compared to the vermis (dragonfly body) in the coronal plane.^6,59,74,75^ However, the ‘dragonfly’ appearance is neither completely sensitive nor specific, as it may be absent in *TSEN54* mutations, present in other PCH types (1, 9, 11 and 16) or present in acquired forms of posterior fossa hypoplasia, especially in prematurity-related injury (Fig. 3).^76-79^

**Table 4:** Range and commonality of neuroimaging attributes by PCH type

Type	1	2	3	4	5	6	7	8	9	10	11	12	13	14	15	16
Age	P-I	P-I	N	P	P	N-I	N	I-C	P-C	I-C	I-C	P	I-C	P	I	P-I
PH	+/−^#^	+/−	+*	+/−*	+/−*	+/−	+*	+*	+*	+/−*	+*	+*	+/−*	+	+*	+
CH	+/−*	+/−	+*	+*	+/−*	+/−*	+*	+*	+*	−^#^	+*	+*	+/−*	+	+*	+/−*
VH	+/−	+/−	+/−	+/−	+/−*	+/−*	+*	+*	+/−	−^#^	+/−	+*	+	+	+*	+/−*
BA	+/−	+/−	−	+*	+/−	+/−	+	-	+/−	-	-	-	+/−*	+/−	+/−	+/−
CA	+/−	+/−	+	+/−	+/−	+/−	+*	+/−	+/−	+/−	+*	-	+	+/−	+/−	+/−
OA	+/−	+/−	-	+/−*	+/−*	+/−	+*	-	+/−	-	-	-	+/−	+/−	-	+/−
WMv	+/−*	+/−	+/−	+/−	-	+/−	+*	+*	+*	+*	+/−	+	+	+	+/−	+/−*
WMm	+/−*	+/−	−^#^	+/−	+/−	+/−	-	-	+/−*	+/−*	−^#^	-	+/−	+/−^#^	+/−	+/−*
CCH	+/−*	+/−	+/−	-	-	+/-	+*	+*	+*	+*	+/−*	+	+	+	+*	+/−*
CeCo	+/−*	+/−	+/−	+/−	+/−	+/−	+/−	-	+/−	+/−	+/−	-	+/−*	+/−	+/−	+/−
ONA	+/−	−^#^	+/−*	-	-	+/−	+/−	-	-	-	+/−^#^	-	-	-	-	-
BG	+/−	+/−	-	+/−	+/−	+/−^#^	-	-	+	-	-	-	+/−	+/−	-	+
Ccyst	+/−	+/−	-	-	-	+/−	-	-	-	-	-	-	-	-	-	-

N, neonate; I, infant; P, prenatal; C, child; PH, pontine hypoplasia; CH, cerebellar hemisphere hypoplasia; VH, vermian hypoplasia; BA, brainstem atrophy; CA, cerebellar atrophy; OA, optic atrophy; WMv, cerebral white matter volume loss or hypoplasia; WMm, white matter myelination deficiency; CCH, corpus callosum hypoplasia or hypogenesis; CeCo, Cerebral cortex volume loss; ONA, olivary nuclear atrophy; BG, basal ganglia volume loss; Ccyst, cerebellar cysts. + = present; - = absent or not reported; +/−, present or absent.

The symbol # denotes that key finding is negative, and * indicates key finding is positive; no special symbol indicates reporting variability and thus not key distinguishing attribute.

Alternative patterns of cerebellar involvement range from diffuse to focal, affecting the vermis and leading to a ‘butterfly’ appearance of the cerebellum in the coronal plane as seen in PCH 3 and PCH 6.^48,80^  *RARS2*-related PCH 6 generally manifests with progressive neurodegeneration, starting with normal volumes in the neonatal period and progressing to moderate or marked diffuse reductions in volume postnatally.^54,80,81^ Reduced white matter diffusion is sometimes seen as well.^81^ As noted, *AMPD2*-related PCH 9 almost always manifests a ‘figure of 8’ appearance of the midbrain in the axial plane due to hypoplastic shortening of the cerebral peduncles.^59,69,79,82,83^ In PCH 10, due to mutations in *CLP1*, there is often only mild pontine hypoplasia with relative or complete sparing of the cerebellum in concert with reduced cerebral white matter volume.^59,84-86^  *TBC1D23*-related PCH 11 is typified by non-progressive but marked pontine hypoplasia, moderate cerebellar hemisphere hypoplasia with associated lateral folial predominant volume loss, mild vermis hypoplasia and marked corpus callosum deficiency ranging from severe hypoplasia to agenesis.^77,78^ Congenital microcephaly with progressive atrophy and simplified gyral pattern typifies PCH 13 due to mutations in *PS51*.^87,88^ Selective basal ganglia hypoplasia and/or atrophy is common in PCH 16.^89,90^

### Emerging PCH-associated genes

Since the end of our review period, at least seven publications have described additional genes implicated in PCH or PCH-like phenotypes. Newly associated genes include *ATAD3A*, *INPP4A* (in two separate reports), *WDR11*—a component of the *FAM91A1* complex, which directly interacts with *TBC1D23*, a previously established PCH gene—as well as *FTH1*, *MPDU1* and *RAF1*.^91–95^ Together, these findings underscore the growing genetic heterogeneity of PCH.^89,90^

## Discussion

### A historically informed comprehensive review

Since 1993, ‘PCH’ has been used to describe a neurodevelopmental disorder affecting the size of the pons and cerebellum with a clinical course that includes severe neurodevelopmental delays or neurodegeneration. Greater resolution of posterior fossa structures has heightened interest in PCH, which has serious implications. Significant clinical, neuroradiographic and genetic overlap among the 26 categories of PCH has likely impeded a neurologist’s ability to have more clarity in prognosis while counselling families. These clinical needs might impact patient care. Therefore, there is a significant need for a clarified PCH definition to guide diagnosis and management—from prenatal screening to post-natal care.

### Considerations in developing a differential

Several features of PCH are also consistent with other prenatal and acquired disorders that lead to substandard growth of the cerebellum and/or pons. In fact, disruptive events of sufficient severity during the course of cerebellar development, including infections, toxins, hypoxic-ischaemic injury, haemorrhage and metabolic disturbances can lead to cerebellar or PCH.^96^ However, PCH is usually not found in isolation. Chromosomal trisomies, such as Down syndrome, often result in underdevelopment of the pons and cerebellum.^97^ Pontocerebellar disruption due to prematurity can lead to a hypoplastic pons and cerebellum, which might not progress to PCH as a disease, but their imaging may be viewed as ‘lesional mimicry.’^98^ Rhombencephalosynapsis, pontine tegmental cap dysplasia, ciliopathies, tubulinopathies and alpha dystroglycanopathies all commonly have some degree of cerebellar and brainstem hypoplasia, though additional structural abnormalities specific to these disorders usually aid in the distinction.^59,99^ Congenital disorders of glycosylation may also present with cerebellar and pontine hypoplasia, often with associated cerebellar volume loss and cortical signal abnormality.^59,99,100^

### PCH mimics

Radiographic and histological features of PCH can lead to diagnostic challenges. For example, a loss of *AGTPBP1* mimics PCH Type 1,^101^ yet this gene is not currently recognized as an aetiology. Similarly, a homozygous deletion in *WDR81* gene^102^ or loss of HEATR5B protein^103^ have been associated with PCH, but not included as a cause. Further, a foetal case of Bainbridge–Ropers Syndrome was later identified as PCH Type 1.^104^ Radiographic features of PCH have also been identified due to mutations in the very low-density lipoprotein receptor [cerebellar ataxia, mental retardation and disequilibrium syndrome 1 (*CAMRQ1*)]^105,106^ and in *DKC1* (Hoyeraal–Hreidarsson Syndrome).^107^ More genes are known to cause similar radiographic features but have not been classified as a PCH disorder, including *PPP2R1A* gene,^108,109^  *PLA2G6*^38^ and *CASK* gene.^110^ An overlap in clinical presentation was also reported in PCH and progressive encephalopathy with oedema, hypsarrhythmia and optic atrophy;^111^ Emanuel syndrome and auditory neuropathy spectrum disorder.^112^

### Integrating neuroimaging and genetic testing towards diagnosing PCH

The diagnosis of PCH is associated with clinical, neuroimaging and genetic characteristics. Among these, neuroimaging is most reliable, especially when followed serially. The clinical course can be variable, and genetic sequencing continues to show an expanding array of aetiologies and associated molecular pathways. Thus, it might be difficult to exclude the diagnosis at time of presentation and with genetic testing alone. Among such variability, imaging characteristics define PCH. Reduced volume of the brainstem and/or cerebellum corresponding to parenchymal hypoplasia with or without associated volume loss is mandatory for the diagnosis. Importantly, serial imaging enhances diagnostic credibility by demonstrating reductions in age-appropriate interval growth or volume loss over time. Greater specificity for the diagnosis is possible when other features are present or develop, including (i) reduced cerebral volume and hypoplastic corpus callosum; (ii) callosal agenesis or dysgenesis; (iii) simplified gyral pattern; (iv) selective volume loss or atrophy of the basal ganglia; (v) deficient myelination; and (vi) olivary hypoplasia, cerebellar cysts and optic hypoplasia and/or volume loss.^35,76^

### Disambiguating PCH towards diagnostic and prognostic clarity

Various terms are used to refer to the diagnosis of PCH, leading to uncertainty and ambiguity within the field. Some classifications with highly overlapping clinical, imaging and genetic features include ‘olivopontocerebellar hypoplasia’, ‘congenital olivopontocerebellar atrophy,’ ‘cerebellar hypoplasia’, ‘pontocerebellar atrophy’, ‘pontine and cerebellar hypoplasia’ and ‘PCCA’, among others. In referring to the disease spectrum as a whole, we propose the term pontocerebellar hypoplasia spectrum disorder (PHSD). We suggest adding the word ‘spectrum’ to represent the range of overlapping features, which are not always pathognomonic for a disease type/subtype.

We agree with efforts to associate types of PCH by genetic aetiology, as in ‘*TSEN* mutation spectrum disorders’ (to include PCH Types 2, 4 and 5),^74^ or to classify PCH Type 6 as a *RARS2*-associated phenotype with early onset mitochondrial encephalopathy.^70^ As such, we propose the introduction of a dyadic naming strategy (e.g. *RARS2*-associated PHSD) to replace the naming conventions that rely on numerical designations (e.g. PCH 1, PCH 2). Traditional naming strategies fail to capture the genetic heterogeneity of these conditions, whereas dyadic naming links the disorder directly to the specific gene involved in its pathogenesis. This enhances clarity in diagnosis and guides targeted research while also resolving inconsistencies in current classification systems by providing a stable framework. When the aetiology is not genetic, abnormal growth of the pons or cerebellum can be defined by the primary aetiology and termed ‘lesional mimicry’.^98^

### Practical recommendations in pre-, peri- and post-natal diagnosis and management

**Figure 4:**
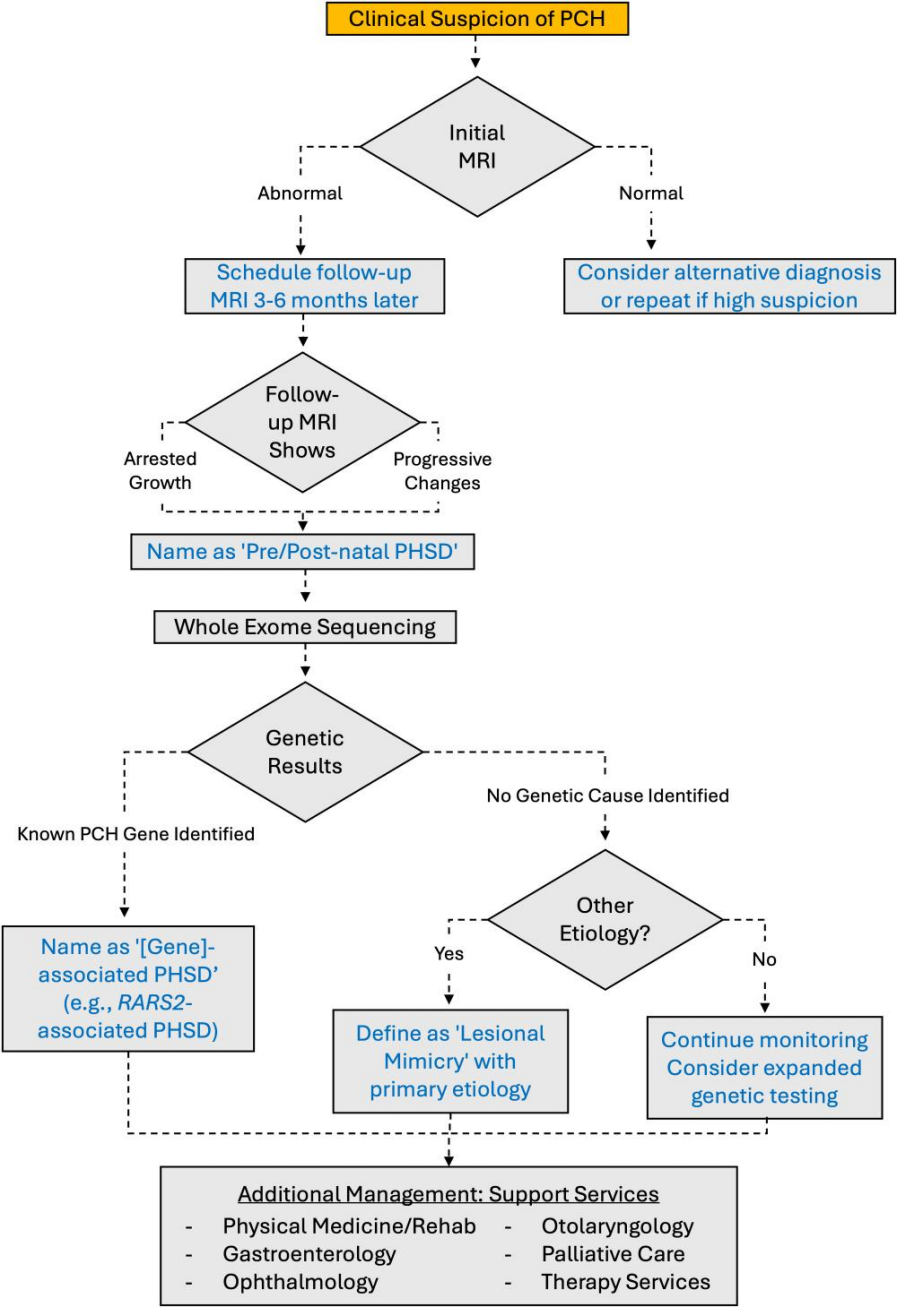
**Proposed diagnostic algorithm for PHSD.** Recommended diagnostic approach for PHSD according to the proposed naming strategy. MRI, magnetic resonance imaging; PCH, pontocerebellar hypoplasia.

Based on our review, we propose the following considerations in diagnosis and management (Fig. 4). Prenatally, concern for PCH is often a by-product of antenatal anatomy ultrasounds during the second trimester. In such cases, initial MR imaging between 20and 25 weeks’ gestation is useful to confirm the findings. Typical MRI evaluation by a paediatric neuroradiologist is needed, and 3D measurements can provide additional supportive information. If the findings are confirmed, repeat imaging between 30 and 34 weeks provides the neurologist and expectant families an opportunity to further consider post-natal management. Follow-up evaluation and imaging, when feasible, is recommended because (i) hypoplasia can become apparent over time due to differences in typical regional growth compared with other brain structures, (ii) later-onset molecular dysfunction can lead to neurodegeneration and regression of volume or (iii) diminished volume can be undetectable at younger gestational ages due to technical limitations in the resolution of imaging. If imaging shows arrested growth or these progressive changes, a diagnosis of pre/post-natal PHSD should be considered. However, in some genetic subtypes, minimal interval change may limit the utility of repeat imaging in the foetal period, which may also be restricted by insurance coverage or access.

Following imaging findings suggestive of PCH, genetic testing should be considered in the prenatal period or postnatally based on family preferences and joint discussions that includes the obstetricians or maternal-foetal specialists and the neurologist. Whole-exome sequencing is recommended because of expected difficulty in distinguishing among PCH types and known genetic heterogeneity; genetic sequencing of individual candidate genes might have lower yield, thus prolonging the diagnostic odyssey. If a genetic cause if identified, the condition should be named as (gene)-associated PHSD.

In the peri- and neonatal period, management can include intensive care to support ventilation and nutrition, additional imaging and referrals to subspecialists and therapists. Infants with PCH can have persistent needs for life-sustaining care and require evaluations for encephalopathy and/or hypotonia. Ultrasound should be obtained initially to evaluate midline, supratentorial structures; greater resolution of the posterior fossa is usually obtained with an MR imaging (preferably non-sedate). As with prenatal management, if clinical diagnosis remains unknown, we recommend follow-up interval imaging, usually 3–6 months after the initial study. When there is a high index of suspicion for PCH based on initial or follow-up imaging, genetic testing is recommended.

Currently, there are no treatments for PCH and affected infants and children are supported symptomatically. Affected individuals may have epilepsy, and their seizures can be refractory to treatment. Greater benefit for affected families might ensue from combination of these supportive measures and prognostic counselling towards anticipating needs and planning care. Clinical consultations often include referral for physical medicine and rehabilitation, gastroenterology, ophthalmology, otolaryngology and palliative care. Frequently, there is a need for longer-term support by physical, occupational and speech therapists, nutritionists and audiologists.

### A much-needed path forward

The majority of PCH studies were retrospective case reports/series, with only a few prospective natural history studies. This persistent gap in the field might bias our prognostic information towards more severely affected children. Thus, there is a pressing need to prospectively consider the clinical, neuroimaging or genetic correlates of long-term outcomes. As a rare, autosomal recessive, neurogenetic disorder, such studies are crucial to systematically characterize PCH by type and correlate types with neurodevelopmental outcomes. Ideally, this would be a multi-centre study, including participants nationally and internationally. In parallel, more basic and translational research is needed to understand the molecular pathophysiology of PCH, as we build a foundation towards developing disease-alleviating (versus. prolonging) therapies.

Overall, we found fewer publications addressing recently described PCH types, especially 10–16. This skew might result from several factors, including (i) true differences in disease frequency; (ii) challenges in diagnosing certain types of PCH due to overlap in clinical, neuroimaging and genetic features; (iii) complexities in differentiating the diseases as they evolve with time and/or (iv) need to accumulate more descriptions with time. Prospective studies could also resolve some of these questions.

## Conclusion

Accurate diagnosis of PCH is usually made with a combination of clinical, neuroradiographic and genetic sequencing data obtained pre- or postnatally through follow-up. To address the growing complexity of PCH classification, we propose adopting the term ‘PHSD’ and implementing a dyadic naming system that links disorders directly to their genetic aetiology. This approach replaces the traditional numerical classification system, which has become increasingly complex with 16 types (categorized as 1–16), 8 subtypes (Types 1 and 2 each have sub-categorization spanning A–F) and over 24 genetic aetiologies. The new system provides greater diagnostic clarity and better reflects our current understanding of the genetic heterogeneity underlying these disorders. There are no treatments for PCH, and neurological care is supportive, personalized and integrated with other medical/therapeutic teams. Moving forward, there is a pressing need for prospective natural history studies to systematically characterize outcomes across the spectrum of PHSD. Future work should also empirically address whether the current categorization system can be reorganized based on a combination of clinical, genetic and neurodiagnostic features.

## Supplementary Material

fcaf298_Supplementary_Data

## Data Availability

Data sharing is not applicable to this article as no new data were created or analysed in this study.

## References

[1] Tamiz AP, Koroshetz WJ, Dhruv NT, Jett DA. A focus on the neural exposome. Neuron. 2022;110(8):1286–1289. 35349785 10.1016/j.neuron.2022.03.019

[2] Barth PG, Vrensen GF, Uylings HB, Oorthuys JW, Stam FC. Inherited syndrome of microcephaly, dyskinesia and pontocerebellar hypoplasia: A systemic atrophy with early onset. J Neurol Sci. 1990;97(1):25–42. 2370559 10.1016/0022-510x(90)90096-6

[3] Albrecht S, Schneider MC, Belmont J, Armstrong DL. Fatal infantile encephalopathy with olivopontocerebellar hypoplasia and micrencephaly. Report of three siblings. Acta Neuropathol. 1993;85(4):394–399. 8480512 10.1007/BF00334450

[4] Budde BS, Namavar Y, Barth PG, et al tRNA splicing endonuclease mutations cause pontocerebellar hypoplasia. Nat Genet. 2008;40(9):1113–1118. 18711368 10.1038/ng.204

[5] Patel MS, Becker LE, Toi A, Armstrong DL, Chitayat D. Severe, fetal-onset form of olivopontocerebellar hypoplasia in three sibs: PCH type 5? Am J Med Genet A. 2006;140(6):594–603. 16470708 10.1002/ajmg.a.31095

[6] Namavar Y, Barth PG, Kasher PR, et al Clinical, neuroradiological and genetic findings in pontocerebellar hypoplasia. Brain. 2011;134(Pt 1):143–156. 20952379 10.1093/brain/awq287 PMC9136852

[7] Barth P . Pontocerebellar hypoplasias: An overview of a group of inherited neurodegenerative disorders with fetal onset. Brain Dev. 1993;15(6):411–422. 8147499 10.1016/0387-7604(93)90080-r

[8] Barth PG, Blennow G, Lenard HG, et al The syndrome of autosomal recessive pontocerebellar hypoplasia, microcephaly, and extrapyramidal dyskinesia (pontocerebellar hypoplasia type 2): Compiled data from 10 pedigrees. Neurology. 1995;45(2):311–317. 7854532 10.1212/wnl.45.2.311

[9] Vogt H, Astwazaturow M. Ueber angeborene kleinhirnerkran kungen mit beitzagen zur entwicklungsgeschichte des kleinhirns. Arch Psychiatr Nervenkrankh. 1912;49:75–203.

[10] Brun R . Zur kenntnis der bildungsfehler des kleinhirns. Epikritische bemerkungen zur entwicklungspathologie, morphologie und klinik der umschriebenen entwicklungshemmungen des neozerebellums. Schweiz Arch Neurol Neurochir Psychiatr. 1917;1:61–123.

[11] Brouwer B . Hypoplasia ponto-neocerebellaris. Psychiatr Neurol (Amst). 1924;6:461–473.

[12] Koster S . Two cases of hypoplasia ponto-neocerebellaris. Acta Psychiatr (Kobenh). 1926;1(1):47–76.

[13] Biemond A . Hypoplasia ponto-neocerebellaris, with malformation of the dentate nucleus. Folia Psychiatr Neurol Neurochir Neerl. 1955;58:2–7. 14353215

[14] Norman RM, Urich H. Cerebellar hypoplasia associated with systemic degeneration in early life. J Neurol Neurosurg Psychiatry. 1958;21(3):159–166. 13576165 10.1136/jnnp.21.3.159 PMC497313

[15] Renbaum P, Kellerman E, Jaron R, et al Spinal muscular atrophy with pontocerebellar hypoplasia is caused by a mutation in the VRK1 gene. Am J Hum Genet. 2009;85(2):281–289. 19646678 10.1016/j.ajhg.2009.07.006 PMC2725266

[16] Weinberg AG, Kirkpatrick JB. Cerebellar hypoplasia in Werdnig-Hoffmann disease. Dev Med Child Neurol. 1975;17(4):511–516. 1158057 10.1111/j.1469-8749.1975.tb03503.x

[17] Goutières F, Aicardi J, Farkas E. Anterior horn cell disease associated with pontocerebellar hypoplasia in infants. J Neurol Neurosurg Psychiatry. 1977;40(4):370–378. 874513 10.1136/jnnp.40.4.370 PMC492704

[18] Peiffer J, Pfeiffer RA. Hypoplasia ponto-neocerebellaris [Hypoplasia ponto-neocerebellaris (author's translation)]. J Neurol. 1977;215(4):241–251. 70516 10.1007/BF00312495

[19] Steiman GS, Rorke LB, Brown MJ. Infantile neuronal degeneration masquerading as Werdnig-Hoffmann disease. Ann Neurol. 1980;8(3):317–324. 7436375 10.1002/ana.410080316

[20] Herrick MK, Strefling AM, Urich H. Intrauterine multisystem atrophy in siblings: A new genetic syndrome? . Acta Neuropathol. 1983;61(1):65–70. 6624387 10.1007/BF00688388

[21] de León GA, Grover WD, Cruz D, A C. Amyotrophic cerebellar hypoplasia: A specific form of infantile spinal atrophy. Acta Neuropathol. 1984;63(4):282–286. 6475488 10.1007/BF00687334

[22] Kawagoe T, Jacob H. Neocerebellar hypoplasia with systemic combined olivo-ponto-dentatal degeneration in a 9-day-old baby: Contribution to the problem of relations between malformation and systemic degeneration in early life. Clin Neuropathol. 1986;5(5):203–208. 3466729

[23] De Caro R, Cortivo P, Crestani C, Parenti A. Bulbopontocerebellar hypoplasia with aplasia of the inferior olivary nucleus. Pathologica. 1987;79(1062):525–531. 3451169

[24] Kamoshita S, Takei Y, Miyao M, Yanagisawa M, Kobayashi S, Saito K. Pontocerebellar hypoplasia associated with infantile motor neuron disease (Norman's disease). Pediatr Pathol. 1990;10(1–2):133–142. 2315227 10.3109/15513819009067102

[25] Barth PG . Inherited progressive disorders of the fetal brain: A field in need of recognition. In: Fukuyama Y, Suzuki Y, Kamoshita S, Casuer P, eds. Fetal and perinatal neurology. Karger; 1992:299–313.

[26] Ryan MM, Cooke-Yarborough CM, Procopis PG, Ouvrier RA. Anterior horn cell disease and olivopontocerebellar hypoplasia. Pediatr Neurol. 2000;23(2):180–184. 11020648 10.1016/s0887-8994(00)00166-1

[27] Ben-Zeev B, Hoffman C, Lev D, et al Progressive cerebellocerebral atrophy: A new syndrome with microcephaly, mental retardation, and spastic quadriplegia. J Med Genet. 2003;40(8):e96. 12920088 10.1136/jmg.40.8.e96 PMC1735539

[28] Boczonadi V, Muller JS, Pyle A, et al EXOSC8 mutations alter mRNA metabolism and cause hypomyelination with spinal muscular atrophy and cerebellar hypoplasia. Nat Commun. 2014;5:4287. 24989451 10.1038/ncomms5287 PMC4102769

[29] Feinstein M, Flusser H, Lerman-Sagie T, et al *VPS53* mutations cause progressive cerebello-cerebral atrophy type 2 (PCCA2). J Med Genet. 2014;51(5):303–308. 24577744 10.1136/jmedgenet-2013-101823

[30] Wan J, Steffen J, Yourshaw M, et al Loss of function of SLC25A46 causes lethal congenital pontocerebellar hypoplasia. Brain. 2016;139(11):2877–2890. 27543974 10.1093/brain/aww212 PMC5840878

[31] Burns DT, Donkervoort S, Muller JS, et al Variants in EXOSC9 disrupt the RNA exosome and result in cerebellar atrophy with spinal motor neuronopathy. Am J Hum Genet. 2018;102(5):858–873. 29727687 10.1016/j.ajhg.2018.03.011 PMC5986733

[32] Somashekar PH, Kaur P, Stephen J, et al Bi-allelic missense variant, p.Ser35Leu in EXOSC1 is associated with pontocerebellar hypoplasia. Clin Genet. 2021;99(4):594–600. 33463720 10.1111/cge.13928 PMC9990822

[33] Sanchez-Albisua I, Frolich S, Barth PG, Steinlin M, Krageloh-Mann I. Natural course of pontocerebellar hypoplasia type 2A. Orphanet J Rare Dis. 2014;9:70. 24886362 10.1186/1750-1172-9-70 PMC4019562

[34] Graham JM Jr, Spencer AH, Grinberg I, et al Molecular and neuroimaging findings in pontocerebellar hypoplasia type 2 (PCH2): Is prenatal diagnosis possible? Am J Med Genet A. 2010;152A(9):2268–2276. 20803644 10.1002/ajmg.a.33579 PMC2931360

[35] Ajibola AJ, Omar SA, Friderici KH. Genetic mutation in pontocerebellar hypoplasia. Clin Genet. Feb. 2010;77(2):197–199. 10.1111/j.1399-0004.2009.01283.x 19807738

[36] Luhl S, Bode H, Schlotzer W, et al Novel homozygous RARS2 mutation in two siblings without pontocerebellar hypoplasia—Further expansion of the phenotypic spectrum. Orphanet J Rare Dis. 2016;11(1):140. 27769281 10.1186/s13023-016-0525-9 PMC5073905

[37] Hady-Cohen R, Ben-Pazi H, Adir V, et al Progressive cerebello-cerebral atrophy and progressive encephalopathy with edema, hypsarrhythmia and optic atrophy may be allelic syndromes. Eur J Paediatr Neurol. 2018;22(6):1133–1138. 30100179 10.1016/j.ejpn.2018.07.003

[38] Gafner M, Michelson M, Yosovich K, Blumkin L, Lerman-Sagie T, Lev D. Infantile onset progressive cerebellar atrophy and anterior horn cell degeneration—A novel phenotype associated with mutations in the PLA2G6 gene. Eur J Med Genet. 2020;63(4):103801. 31689548 10.1016/j.ejmg.2019.103801

[39] Nakamura K, Nishiyama K, Kodera H, et al A de novo CASK mutation in pontocerebellar hypoplasia type 3 with early myoclonic epilepsy and tetralogy of Fallot. Brain Dev. 2014;36(3):272–273. 23623288 10.1016/j.braindev.2013.03.007

[40] Simonati A, Cassandrini D, Bazan D, Santorelli FM. TSEN54 mutation in a child with pontocerebellar hypoplasia type 1. Acta Neuropathol. 2011;121(5):671–673. 21468723 10.1007/s00401-011-0823-1

[41] van Dijk T, Rudnik-Schoneborn S, Senderek J, et al Pontocerebellar hypoplasia with spinal muscular atrophy (PCH1): Identification of SLC25A46 mutations in the original Dutch PCH1 family. Brain. 2017;140(8):e46. 28637197 10.1093/brain/awx147

[42] Norman RM . Cerebellar hypoplasia in Werdnig-Hoffmann disease. Arch Dis Child. 1961;36(185):96–101. 13729575 10.1136/adc.36.185.96 PMC2012675

[43] Vinograd-Byk H, Sapir T, Cantarero L, et al The spinal muscular atrophy with pontocerebellar hypoplasia gene VRK1 regulates neuronal migration through an amyloid-β precursor protein-dependent mechanism. J Neurosci. 2015;35(3):936–942. 25609612 10.1523/JNEUROSCI.1998-14.2015 PMC6605533

[44] Wan J, Yourshaw M, Mamsa H, et al Mutations in the RNA exosome component gene EXOSC3 cause pontocerebellar hypoplasia and spinal motor neuron degeneration. Nat Genet. 2012;44(6):704–708. 22544365 10.1038/ng.2254 PMC3366034

[45] Agamy O, Ben Zeev B, Lev D, et al Mutations disrupting selenocysteine formation cause progressive cerebello-cerebral atrophy. Am J Hum Genet. 2010;87(4):538–544. 20920667 10.1016/j.ajhg.2010.09.007 PMC2948803

[46] Breuss MW, Sultan T, James KN, et al Autosomal-recessive mutations in the tRNA splicing endonuclease subunit TSEN15 cause pontocerebellar hypoplasia and progressive microcephaly. Am J Hum Genet. 2016;99(1):228–235. 27392077 10.1016/j.ajhg.2016.05.023 PMC5005448

[47] Rajab A, Mochida GH, Hill A, et al A novel form of pontocerebellar hypoplasia maps to chromosome 7q11-21. Neurology. 2003;60(10):1664–1667. 12771259 10.1212/01.wnl.0000068548.58498.41

[48] Ahmed MY, Chioza BA, Rajab A, et al Loss of PCLO function underlies pontocerebellar hypoplasia type III. Neurology. 2015;84(17):1745–1750. 25832664 10.1212/WNL.0000000000001523 PMC4424132

[49] Falck J, Bruns C, Hoffmann-Conaway S, et al Loss of Piccolo function in rats induces cerebellar network dysfunction and pontocerebellar hypoplasia type 3-like phenotypes. J Neurosci. 2020;40(14):2943–2959. 32122952 10.1523/JNEUROSCI.2316-19.2020 PMC7117892

[50] Mahbubul Huq AH, Nigro MA. XY sex reversal and a nonprogressive neurologic disorder: A new syndrome? Pediatr Neurol. 2000;23(4):357–360. 11068172 10.1016/s0887-8994(00)00200-9

[51] Zafeiriou DI, Ververi A, Tsitlakidou A, Anastasiou A, Vargiami E. Recurrent episodes of rhabdomyolysis in pontocerebellar hypoplasia type 2. Neuromuscul Disord. 2013;23(2):116–119. 23177318 10.1016/j.nmd.2012.08.004

[52] Jacob FD, Hasal S, Goez HR. Pontocerebellar hypoplasia type 3 with severe vitamin A deficiency. Pediatr Neurol. 2011;44(2):147–149. 21215917 10.1016/j.pediatrneurol.2010.09.002

[53] Jinnou H, Okanishi T, Enoki H, Ohki S. Pontocerebellar hypoplasia type 3 with tetralogy of Fallot. Brain Dev. 2012;34(5):392–395. 21880448 10.1016/j.braindev.2011.07.011

[54] Ngoh A, Bras J, Guerreiro R, et al RARS2 mutations in a sibship with infantile spasms. Epilepsia. 2016;57(5):e97–e102. 27061686 10.1111/epi.13358 PMC4864753

[55] Abreu NJ, Koboldt DC, Gastier-Foster JM, Dave-Wala A, Flanigan KM, Waldrop MA. Homozygous variants in AMPD2 and COL11A1 lead to a complex phenotype of pontocerebellar hypoplasia type 9 and stickler syndrome type 2. Am J Med Genet A. 2020;182(3):557–560. 31833174 10.1002/ajmg.a.61452

[56] Saugier-Veber P, Marguet F, Vezain M, et al Pontocerebellar hypoplasia with rhombencephalosynapsis and microlissencephaly expands the spectrum of PCH type 1B. Eur J Med Genet. 2020;63(4):103814. 31770597 10.1016/j.ejmg.2019.103814

[57] Wuest A, Surbek D, Wiest R, et al Enlarged posterior fossa on prenatal imaging: Differential diagnosis, associated anomalies and postnatal outcome. Acta Obstet Gynecol Scand. 2017;96(7):837–843. 28295149 10.1111/aogs.13131

[58] Goasdoue P, Rodriguez D, Moutard ML, Robain O, Lalande G, Adamsbaum C. Pontoneocerebellar hypoplasia: Definition of MR features. Pediatr Radiol. 2001;31(9):613–618. 11511999 10.1007/s002470100507

[59] van Dijk T, Baas F, Barth PG, Poll-The BT. What's new in pontocerebellar hypoplasia? An update on genes and subtypes. Orphanet J Rare Dis. 2018;13(1):92. 29903031 10.1186/s13023-018-0826-2 PMC6003036

[60] Pacheva IH, Todorov T, Ivanov I, et al TSEN54 gene-related pontocerebellar hypoplasia type 2 could mimic dyskinetic cerebral palsy with severe psychomotor retardation. Front Pediatr. 2018;6:1. 29410950 10.3389/fped.2018.00001 PMC5787054

[61] van Dijk T, Ferdinandusse S, Ruiter JPN, et al Biallelic loss of function variants in COASY cause prenatal onset pontocerebellar hypoplasia, microcephaly, and arthrogryposis. Eur J Hum Genet. 2018;26(12):1752–1758. 30089828 10.1038/s41431-018-0233-0 PMC6244412

[62] Leibovitz Z, Shkolnik C, Haratz KK, Malinger G, Shapiro I, Lerman-Sagie T. Assessment of fetal midbrain and hindbrain in mid-sagittal cranial plane by three-dimensional multiplanar sonography. Part 2: Application of nomograms to fetuses with posterior fossa malformations. Ultrasound Obstet Gynecol. 2014;44(5):581–587. 24478245 10.1002/uog.13312

[63] Griffiths PD, Brackley K, Bradburn M, et al Anatomical subgroup analysis of the MERIDIAN cohort: Posterior fossa abnormalities. Ultrasound Obstet Gynecol. 2017;50(6):745–752. 28397323 10.1002/uog.17485

[64] Kyriakopoulou V, Vatansever D, Davidson A, et al Normative biometry of the fetal brain using magnetic resonance imaging. Brain Struct Funct. 2017;222(5):2295–2307. 27885428 10.1007/s00429-016-1342-6 PMC5504265

[65] Scott JA, Hamzelou KS, Rajagopalan V, et al 3D morphometric analysis of human fetal cerebellar development. Cerebellum. 2012;11(3):761–770. 22198870 10.1007/s12311-011-0338-2 PMC3389138

[66] Andescavage NN, du Plessis A, McCarter R, et al Complex trajectories of brain development in the healthy human fetus. Cereb Cortex. 2017;27(11):5274–5283. 27799276 10.1093/cercor/bhw306 PMC6074870

[67] da Silva NA Jr, Vassallo J, Sarian LO, Cognard C, Sevely A. Magnetic resonance imaging of the fetal brain at 3 tesla: Preliminary experience from a single series. Medicine (Baltimore). 2018;97(40):e12602. 30290631 10.1097/MD.0000000000012602 PMC6200506

[68] Priego G, Barrowman NJ, Hurteau-Miller J, Miller E. Does 3T fetal MRI improve image resolution of normal brain structures between 20 and 24 weeks’ gestational age? AJNR Am J Neuroradiol. 2017;38(8):1636–1642. 28619840 10.3174/ajnr.A5251 PMC7960432

[69] Kortum F, Jamra RA, Alawi M, et al Clinical and genetic spectrum of AMPD2-related pontocerebellar hypoplasia type 9. Eur J Hum Genet. 2018;26(5):695–708. 29463858 10.1038/s41431-018-0098-2 PMC5945775

[70] van Dijk T, van Ruissen F, Jaeger B, et al RARS2 mutations: Is pontocerebellar hypoplasia type 6 a mitochondrial encephalopathy? JIMD Rep. 2017;33:87–92. 27683254 10.1007/8904_2016_584 PMC5413457

[71] Fiori S, Poretti A, Pannek K, et al Diffusion tractography biomarkers of pediatric cerebellar hypoplasia/atrophy: Preliminary results using constrained spherical deconvolution. AJNR Am J Neuroradiol. 2016;37(5):917–923. 26659337 10.3174/ajnr.A4607 PMC7960301

[72] Ivanov I, Atkinson D, Litvinenko I, et al Pontocerebellar hypoplasia type 1 for the neuropediatrician: Genotype-phenotype correlations and diagnostic guidelines based on new cases and overview of the literature. Eur J Paediatr Neurol. 2018;22(4):674–681. 29656927 10.1016/j.ejpn.2018.03.011

[73] Rudnik-Schoneborn S, Senderek J, Jen JC, et al Pontocerebellar hypoplasia type 1: Clinical spectrum and relevance of EXOSC3 mutations. Neurology. 2013;80(5):438–446. 23284067 10.1212/WNL.0b013e31827f0f66 PMC3590055

[74] Namavar Y, Chitayat D, Barth PG, et al TSEN54 mutations cause pontocerebellar hypoplasia type 5. Eur J Hum Genet. Jun. 2011;19(6):724–726. 10.1038/ejhg.2011.8 PMC3110057 21368912

[75] Namavar Y, Eggens VRC, Barth PG, et al TSEN54-related pontocerebellar hypoplasia. In: Adam MP, Ardinger HH, Pagon RA, eds. Genereviews. University of Washington, Seattle; 1993. 20301773

[76] Rudnik-Schoneborn S, Barth PG, Zerres K. Pontocerebellar hypoplasia. Am J Med Genet C Semin Med Genet. 2014;166C(2):173–183. 24924738 10.1002/ajmg.c.31403

[77] Marin-Valencia I, Gerondopoulos A, Zaki MS, et al Homozygous mutations in TBC1D23 lead to a non-degenerative form of pontocerebellar hypoplasia. Am J Hum Genet. 2017;101(3):441–450. 28823706 10.1016/j.ajhg.2017.07.015 PMC5590949

[78] Ivanova EL, Mau-Them FT, Riazuddin S, et al Homozygous truncating variants in TBC1D23 cause pontocerebellar hypoplasia and alter cortical development. Am J Hum Genet. 2017;101(3):428–440. 28823707 10.1016/j.ajhg.2017.07.010 PMC5590842

[79] Akizu N, Cantagrel V, Schroth J, et al AMPD2 regulates GTP synthesis and is mutated in a potentially treatable neurodegenerative brainstem disorder. Cell. 2013;154(3):505–517. 23911318 10.1016/j.cell.2013.07.005 PMC3815927

[80] Glamuzina E, Brown R, Hogarth K, et al Further delineation of pontocerebellar hypoplasia type 6 due to mutations in the gene encoding mitochondrial arginyl-tRNA synthetase, RARS2. J Inherit Metab Dis. 2012;35(3):459–467. 22086604 10.1007/s10545-011-9413-6

[81] Zhang J, Zhang Z, Zhang Y, Wu Y. Distinct magnetic resonance imaging features in a patient with novel RARS2 mutations: A case report and review of the literature. Exp Ther Med. 2018;15(1):1099–1104. 29434700 10.3892/etm.2017.5491 PMC5772945

[82] Marsh AP, Lukic V, Pope K, et al Complete callosal agenesis, pontocerebellar hypoplasia, and axonal neuropathy due to AMPD2 loss. Neurol Genet. 2015;1(2):e16. 27066553 10.1212/NXG.0000000000000014 PMC4807911

[83] Marsh APL, Novarino G, Lockhart PJ, Leventer RJ. CUGC for pontocerebellar hypoplasia type 9 and spastic paraplegia-63. Eur J Hum Genet. 2019;27(1):161–166. 30089829 10.1038/s41431-018-0231-2 PMC6303251

[84] Wafik M, Taylor J, Lester T, Gibbons RJ, Shears DJ. 2 new cases of pontocerebellar hypoplasia type 10 identified by whole exome sequencing in a Turkish family. Eur J Med Genet. 2018;61(5):273–279. 29307788 10.1016/j.ejmg.2018.01.002

[85] Karaca E, Weitzer S, Pehlivan D, et al Human CLP1 mutations alter tRNA biogenesis, affecting both peripheral and central nervous system function. Cell. 2014;157(3):636–650. 24766809 10.1016/j.cell.2014.02.058 PMC4146440

[86] Schaffer AE, Eggens VR, Caglayan AO, et al CLP1 founder mutation links tRNA splicing and maturation to cerebellar development and neurodegeneration. Cell. 2014;157(3):651–663. 24766810 10.1016/j.cell.2014.03.049 PMC4128918

[87] Uwineza A, Caberg JH, Hitayezu J, et al VPS51 biallelic variants cause microcephaly with brain malformations: A confirmatory report. Eur J Med Genet. 2019;62(8):103704. 31207318 10.1016/j.ejmg.2019.103704

[88] Gershlick DC, Ishida M, Jones JR, Bellomo A, Bonifacino JS, Everman DB. A neurodevelopmental disorder caused by mutations in the VPS51 subunit of the GARP and EARP complexes. Hum Mol Genet. 2019;28(9):1548–1560. 30624672 10.1093/hmg/ddy423 PMC6489419

[89] Appelhof B, Wagner M, Hoefele J, et al Pontocerebellar hypoplasia due to bi-allelic variants in MINPP1. Eur J Hum Genet. 2021;29(3):411–421. 33168985 10.1038/s41431-020-00749-x PMC7940488

[90] Ucuncu E, Rajamani K, Wilson MSC, et al MINPP1 prevents intracellular accumulation of the chelator inositol hexakisphosphate and is mutated in pontocerebellar hypoplasia. Nat Commun. 2020;11(1):6087. 33257696 10.1038/s41467-020-19919-y PMC7705663

[91] Zhao L, Deng H, Yang Q, et al FAM91A1-TBC1D23 complex structure reveals human genetic variations susceptible for PCH. Proc Natl Acad Sci U S A. 2023;120(45):e2309910120. 37903274 10.1073/pnas.2309910120 PMC10636324

[92] Shieh JT, Tintos-Hernandez JA, Murali CN, et al Heterozygous nonsense variants in the ferritin heavy-chain gene FTH1 cause a neuroferritinopathy. HGG Adv. 2023;4(4):100236. 37660254 10.1016/j.xhgg.2023.100236 PMC10510067

[93] Darouich S, Bellamine H, Khamassi I. Severe ciliopathy-like phenotype in an infant with a novel MPDU1 missense variant. Pediatr Dev Pathol. 2023;26(2):161–165. 36755425 10.1177/10935266231151773

[94] Darouich S, Chakroun AS, Bellamine H, Khamassi I. A severe clinicopathologic phenotype of RAF1 Ser257Leu neomutation in a preterm infant without cardiac anomaly. Am J Med Genet A. 2023;191(2):630–633. 36333975 10.1002/ajmg.a.63035

[95] Hecher L, Harms FL, Lisfeld J, Alawi M, Denecke J, Kutsche K. INPP4A-related genetic and phenotypic spectrum and functional relevance of subcellular targeting of INPP4A isoforms. Neurogenetics. 2023;24(2):79–93. 36653678 10.1007/s10048-023-00709-9

[96] Bosemani T, Poretti A. Cerebellar disruptions and neurodevelopmental disabilities. Semin Fetal Neonatal Med. 2016;21(5):339–348. 27184462 10.1016/j.siny.2016.04.014

[97] Shiohama T, Levman J, Baumer N, Takahashi E. Structural magnetic resonance imaging-based brain morphology study in infants and toddlers with down syndrome: The effect of comorbidities. Pediatr Neurol. 2019;100:67–73. 31036426 10.1016/j.pediatrneurol.2019.03.015 PMC6755072

[98] Steinlin M, Klein A, Haas-Lude K, et al Pontocerebellar hypoplasia type 2: Variability in clinical and imaging findings. Eur J Paediatr Neurol. 2007;11(3):146–152. 17320436 10.1016/j.ejpn.2006.11.012

[99] Barkovich AJ, Raybaud C. Pediatric neuroimaging. 5th edn. Wolters Kluwer Health/Lippincott Williams & Wilkins; 2012:xvii, 1125.

[100] Feraco P, Mirabelli-Badenier M, Severino M, et al The shrunken, bright cerebellum: A characteristic MRI finding in congenital disorders of glycosylation type 1a. AJNR Am J Neuroradiol. 2012;33(11):2062–2067. 22723063 10.3174/ajnr.A3151 PMC7965601

[101] Sheffer R, Gur M, Brooks R, et al Biallelic variants in AGTPBP1, involved in tubulin deglutamylation, are associated with cerebellar degeneration and motor neuropathy. Eur J Hum Genet. 2019;27(9):1419–1426. 30976113 10.1038/s41431-019-0400-y PMC6777529

[102] Kalmar T, Szakszon K, Maroti Z, et al A novel homozygous frameshift WDR81 mutation associated with microlissencephaly, corpus callosum agenesis, and pontocerebellar hypoplasia. J Pediatr Genet. 2021;10(2):159–163. 33996189 10.1055/s-0040-1712916 PMC8110363

[103] Ghosh SG, Breuss MW, Schlachetzki Z, et al Biallelic hypomorphic mutations in HEATR5B, encoding HEAT repeat-containing protein 5B, in a neurological syndrome with pontocerebellar hypoplasia. Eur J Hum Genet. 2021;29(6):957–964. 33824466 10.1038/s41431-021-00832-x PMC8187379

[104] Bacrot S, Mechler C, Talhi N, et al Whole exome sequencing diagnoses the first fetal case of Bainbridge-Ropers syndrome presenting as pontocerebellar hypoplasia type 1. Birth Defects Res. 2018;110(6):538–542. 29316359 10.1002/bdr2.1191

[105] Sonmez FM, Gleeson JG, Celep F, Kul S. The very low density lipoprotein receptor-associated pontocerebellar hypoplasia and dysmorphic features in three Turkish patients. J Child Neurol. 2013;28(3):379–383. 22532556 10.1177/0883073812441065 PMC4442636

[106] Wilker M, Christen HJ, Schuster S, Abicht A, Boltshauser E. VLDLR-associated pontocerebellar hypoplasia with nonprogressive congenital ataxia and a diagnostic neuroimaging pattern. Neuropediatrics. 2019;50(6):404–405. 31261436 10.1055/s-0039-1688953

[107] Dehmel M, Brenner S, Suttorp M, et al Novel mutation in the DKC1 gene: Neonatal Hoyeraal-Hreidarsson syndrome as a rare differential diagnosis in pontocerebellar hypoplasia, primary microcephaly, and progressive bone marrow failure. Neuropediatrics. 2016;47(3):182–186. 26951492 10.1055/s-0036-1578799

[108] Ruxmohan S, Quinonez J, Yadav RS, Shrestha S, Poudel S, Stein JD. Refractory epilepsy in a toddler with PPP2R1A gene mutation and congenital hydrocephalus. Cureus. 2021;13(11):e19988. 34984142 10.7759/cureus.19988 PMC8715662

[109] Wallace A, Caruso P, Karaa A. A newborn with severe ventriculomegaly: Expanding the PPP2R1A gene mutation phenotype. J Pediatr Genet. 2019;8(4):240–243. 31687265 10.1055/s-0039-1692414 PMC6824891

[110] Shelby ES, Lupu OT, Axente M, et al New VOUS in CASK gene correlating with the MICPCH phenotype. Maedica (Bucur). 2021;16(1):135–139. 34221169 10.26574/maedica.2020.16.1.135 PMC8224713

[111] Nevanlinna V, Konovalova S, Ceulemans B, et al A patient with pontocerebellar hypoplasia type 6: Novel RARS2 mutations, comparison to previously published patients and clinical distinction from PEHO syndrome. Eur J Med Genet. 2020;63(3):103766. 31536827 10.1016/j.ejmg.2019.103766

[112] Desai S, Desai T. Prenatal diagnosis of pontocerebellar hypoplasia associated with rare syndromes: Expanding the genetic and phenotypic spectrum. Ultrasound Obstet Gynecol. 2020;57(3):498–499. 10.1002/uog.22038 32250494

