# Supplementary material for: Pontocerebellar hypoplasia: a review from 1912 to 2022
Source: Brain Commun. 2025 Aug 17;7(5):fcaf298. doi: 10.1093/braincomms/fcaf298 (PMC12422213; doi:10.1093/braincomms/fcaf298)
Supplement: fcaf298_Supplementary_Data [file fcaf298_supplementary_data.pdf]

SUPPLEMENTARY MATERIALS

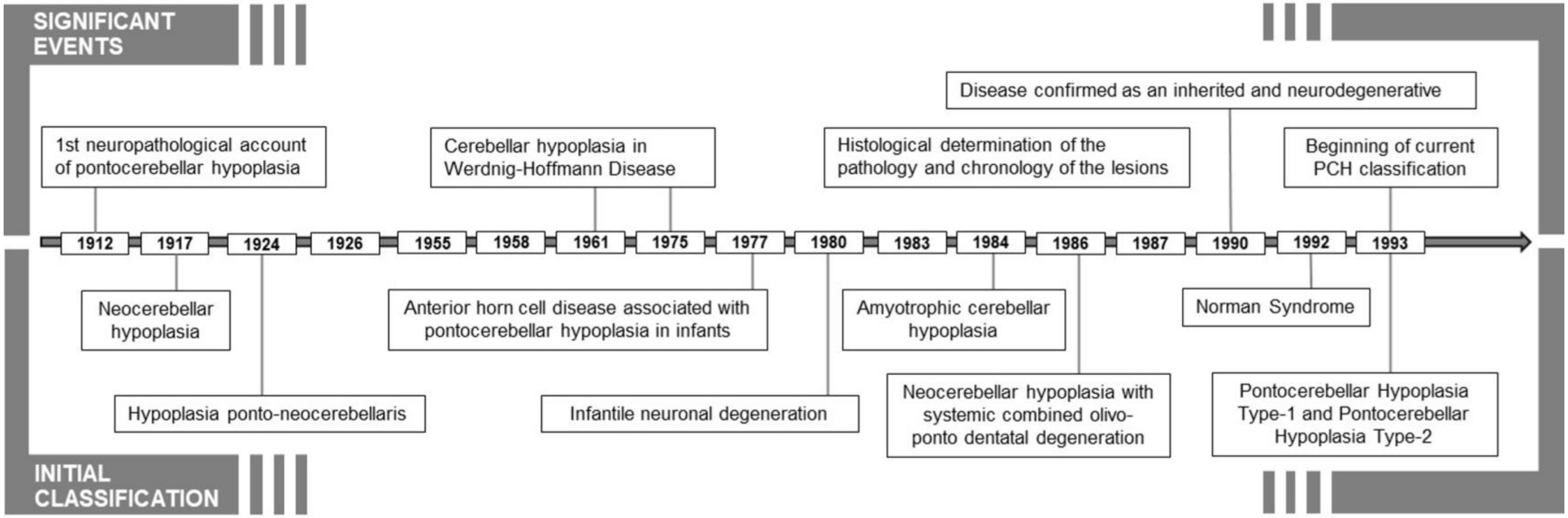

Supplementary Figure 1. Chronological Representation of Milestones in the PCH Literature. From 1912 to 1993 several significant events contributed to the formation of PCH as a diagnosis. Prior to 1993 several case reports/ series described the clinical and pathophysiological manifestations of the disease. Different eponyms were given based on prominent and distinguishing disease features. In 1993, Barth et al. coined the term PCH 1 and 2, thus associating clinical, histopathological and radiographic features into a disorder.[1]

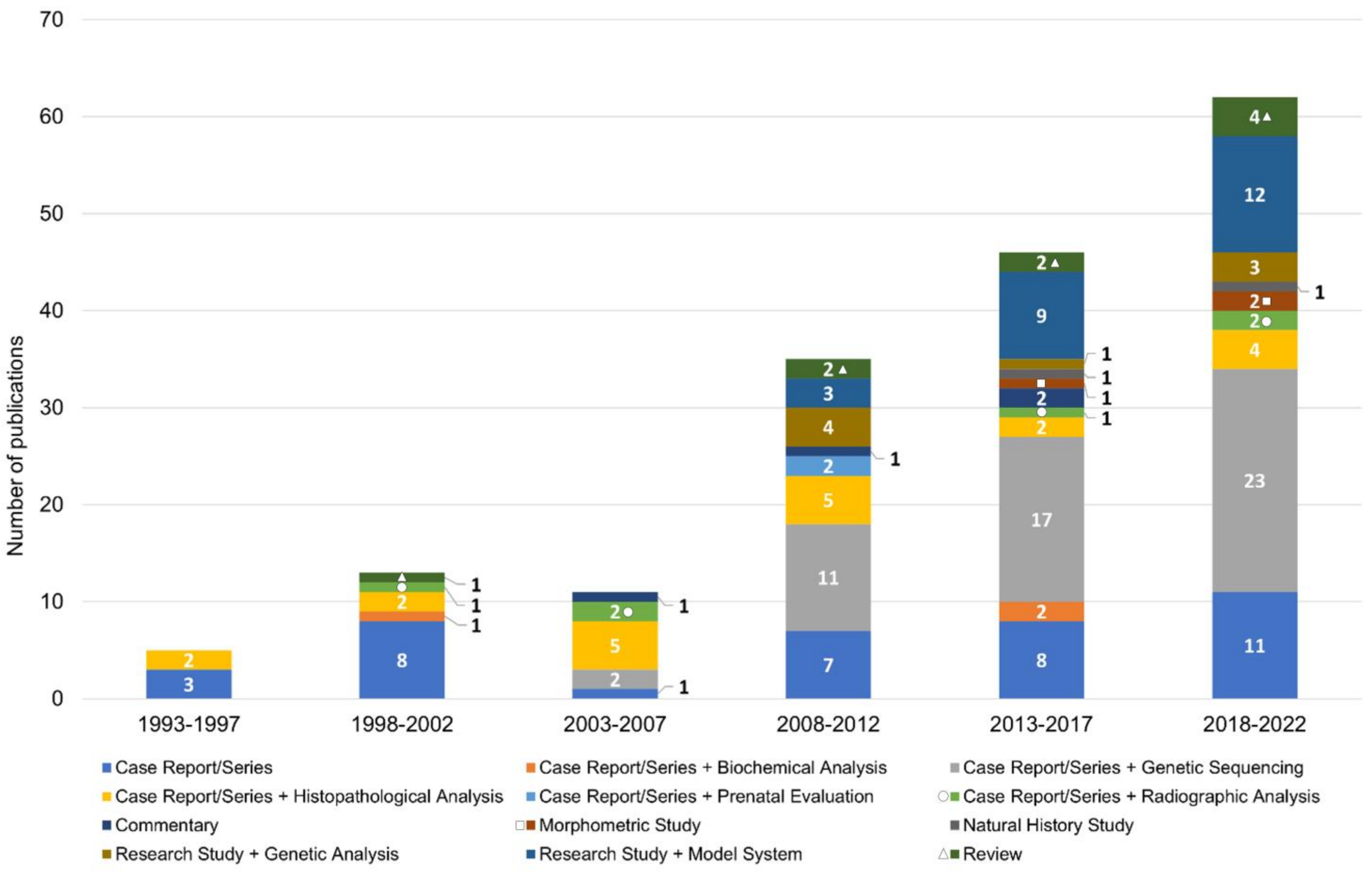

Supplementary Figure 2. Chronological Overview of the PCH Literature. This graph demonstrates the summed number and type of publications sorted by 5-year increments between 1993-2022. The literature was purposely categorized into case report/series that give insight into patient's clinical course, and furthermore by the publication's additional contribution to PCH histopathology, biochemistry, prenatal evaluation, genetics and neuroimaging. We also report commentaries and reviews that summarize the field, natural history and morphometric studies, and research studies focusing on either genetics or disease animal model system. Aside from commentaries/ reviews, each publication focuses on a limited range of PCH types or subtypes prompting further investigation. To support broader accessibility, we have incorporated shape-based annotations: ■ (square for red), ● (circle for light green), and ▲ (triangle for dark green).

PCH Phenotype	Locus	IHT	Gene	Gene function	First Report in PCH	Gene Identification in PCH	Clinical features	PCH Animal Models
**Pontocerebellar Hypoplasia Type 1**								
Type-1A	14q32.2	AR	*VRK1*	Neuronal migration	Norman (1961)[2]	Renbaum et al. (2009)[3]	Onset prenatal to late infancy, SMA phenotype, polyneuropathy	**Mouse:** Vinograd-Byk et al. (2015)[4]
Type-1B	9p13.2	AR	*EXOSC3*	mRNA degradation	Ryan et al. (2000)[5]	Wan et al. (2012)	Neonatal onset, early neonatal apnea, oculomotor apraxia (+/−), optic atrophy (+/−), tongue fasciculations, contractures, axonal motor neuropathy	none
Type-1C	13q13.3	AR	*EXOSC8*	mRNA degradation	Boczonadi et al. (2014)[7]	Boczonadi et al. (2014)[7]	Onset in first months of life, respiratory failure, contractures, SMA phenotype, atrophy, hearing impairment	**Zebrafish**: Boczonadi et al. (2014)[7]
Type-1D	4q27	AR	*EXOSC9*	mRNA degradation	Burns et al. (2018)[8]	Burns et al. (2018)[8]	Onset at birth to early infancy, reduced fetal movements, early neonatal apnea, contractures, fasciculations, impaired pursuit, axonal motor neuronopathy	**Zebrafish:** Burns et al. (2018)[8]
Type-1E	5q22.1	AR	*SLC25A46*	Mitochondrial fission/ fusion	Wan et al. (2016)[9]	Wan et al. (2016)[9]	Prenatal onset, neonatal lethal, polyhydramnios, early neonatal apnea, contractures, optic atrophy (+/−), polyneuropathy	**Zebrafish**: Wan et al. (2016)[9]
Type-1F **#**	10q24.1	AR	*EXOSC1*	mRNA degradation	Somashekar et al. (2021)[10]	Somashekar et al. (2021)[10]	Onset in infancy, developmental delays, blue sclera, microcephaly, dysmorphic facies, hypotonia, diminished reflexes	none
**Pontocerebellar Hypoplasia Type 2**								
Type-2A	17q25.1	AR	*TSEN54*	tRNA splicing	Barth et al. (1990)[11]	Budde et al. (2008)[12]	Onset at birth, impaired swallowing, central visual impairment, hypertonia at birth, extrapyramidal dyskinesia	none
Type-2B	3p25.2	AR	*TSEN2*	tRNA splicing	Barth et al. (1990)[11]	Namavar et al. (2011)[13]	Onset at birth, central visual impairment, hyperkinetic involuntary movements	none
Type-2C **#**	19q13.42	AR	*TSEN34*	tRNA splicing	Barth et al. (1990)[11]	Budde et al. (2008)[12]	Onset at birth, extrapyramidal dyskinesia	none
Type-2D	4p15.2	AR	*SEPSECS*	Selenocysteine synthesis catalyst	Ben-Zeev et al. (2003)[14]	Agamy et al. (2010)[15]	Onset in infancy, contractures, sleep disturbances, irritability, edema of face and limbs, polyneuropathy, optic atrophy (+/−)	none
Type-2E	17p13.3	AR	*VPS53*	Retrograde transport of endosomes to Golgi	Feinstein et al. (2014)[16]	Feinstein et al. (2014)[16]	Onset in infancy, progressive, failure to thrive, gaze-evoked nystagmus (+/−), optic atrophy (+/−), distal limb edema (+/−)	none
Type-2F	1q25.3	AR	*TSEN15*	tRNA splicing	Barth et al. (1990)[11]	Breuss et al. (2016)[17]	Onset at birth, poor or absent fixation	none
**Pontocerebellar Hypoplasia Type 3**								
Type-3 **#**	7q21.11	AR	*PCLO*	Regulation of synaptic protein/vesicle formation	Rajab et al. (2003)[18]	Ahmed et al. (2015)[19]	Onset at birth, neonatal hypotonia, optic atrophy	**Rat:** Falck et al. (2020)[20]
**Pontocerebellar Hypoplasia Type 4**								
Type-4	17q25.1	AR	*TSEN54*	tRNA splicing	Albrecht et al. (1993)[21]	Budde et al. (2008)[12]	Prenatal onset, death in infancy, polyhydramnios, early neonatal apnea, contractures	none
**Pontocerebellar Hypoplasia Type 5**								
Type-5 **#**	17q25.1	AR	*TSEN54*	tRNA splicing	Patel et al. (2006)[22]	Namavar et al. (2011)[13]	Prenatal onset, death in neonatal period, seizure including seizure-like activity in utero starting around 18 weeks gestation, polyhydramnios, early neonatal apnea	none

Supplementary Table 1. PCH Categorization. Complete PCH categorization, listed by type/subtype, locus, associated genetic etiology, protein function, inheritance pattern, OMIM IDs, publications first reporting the disease and associated genetic etiology, and availability of translational/model research systems. A hashtag (**#**) indicates a provisional relationship between the phenotype and gene per OMIM. Abbreviations: IHT = Inheritance, AR = Autosomal Recessive.

PCH Phenotype	Locus	IHT	Gene	Gene function	First Report in PCH	Gene Identification in PCH	Clinical features	PCH Animal Models
**Pontocerebellar Hypoplasia Type 6**								
Type-6	6q15	AR	*RARS2*	Arginyl-tRNA synthetase	Edvardson et al. (2007)[23]	Edvardson et al. (2007)[23]	Neonatal onset, failure to thrive, dysconjugate eye movements, optic atrophy (+/−), apneic episodes, increased serum/CSF lactate	none
**Pontocerebellar Hypoplasia Type 7**								
Type-7	1p34.1	AR	*TOE1*	Regulation of cell growth	Mahbubul Huq et al. (2000)[24]	Lardelli et al. (2017)[25]	Onset at birth, ocular motor apraxia (+/−), optic atrophy (+/−), abnormal breathing pattern, ambiguous genitalia, poor spontaneous movements, myoclonus	**Zebrafish**: Lardelli et al. (2017)[25]
**Pontocerebellar Hypoplasia Type 8**								
Type-8	16q24.3	AR	*CHMP1A*	Regulation of *INK4A*	Mochida et al. (2012)[26]	Mochida et al. (2012)[26]	Onset at birth, cortical visual impairment (+/−), choreiform movements	**Zebrafish & Mouse:** Mochida et al. (2012)[26]
**Pontocerebellar Hypoplasia Type 9**								
Type-9	1p13.3	AR	*AMPD2*	Regulation of GTP synthesis	Akizu et al. (2013)[27]	Akizu et al. (2013)[27]	Onset at birth or early infancy, cortical blindness, absent development, axonal neuropathy (+/−)	**Yeast & Mouse:** Akizu et al. (2013)[27]
**Pontocerebellar Hypoplasia Type 10**								
Type-10	11q12.1	AR	*CLP1*	tRNA splicing	Karaca et al. (2014)[28] Schaffer et al. (2014)[29]	Karaca et al. (2014)[28] Schaffer et al. (2014)[29]	Onset at birth, polyneuropathy, microcephaly, developmental delays, intractable epilepsy, dysmorphic features	**Mouse:** Hanada et al. (2013)[30], Karaca et al. (2014)[28], Monaghan et al. (2021)[31], Morisaki et al. (2021)[32] **Zebrafish**: Schaffer et al. (2014)[29]
**Pontocerebellar Hypoplasia Type 11**								
Type-11	3q12.1-q12.2	AR	*TBC1D23*	Intracellular vesicle transport	Ivanova et al. (2017)[33] Marin-Valencia et al. (2017)[34]	Ivanova et al. (2017)[33] Marin-Valencia et al. (2017)[34]	Onset in early infancy, non-progressive, autistic features, attention deficit-hyperactivity, hearing impairment (+/−)	**Mouse**: Ivanova et al. (2017)[33] **Zebrafish**: Marin-Valencia et al. (2017)[34]
**Pontocerebellar Hypoplasia Type 12**								
Type-12	17q21.2	AR	*COASY*	CoA synthesis	Van Dijk et al. (2018)[35]	Van Dijk et al. (2018)[35]	Prenatal onset, death in infancy, contractures, polyhydramnios (+/−)	none
**Pontocerebellar Hypoplasia Type 13**								
Type-13	11q13.1	AR	*VPS51*	Retrograde transport of endosomes to Golgi	Gershlick et al. (2019)[36] Uwineza et al. (2019)[37]	Gershlick et al. (2019)[36] Uwineza et al. (2019)[37]	Onset in infancy, failure to thrive, cortical visual impairment, hepatomegaly (+/−)	none
**Pontocerebellar Hypoplasia Type 14**								
Type-14	6p21.2	AR	*PPIL1*	Gene expression and regulation	Chai et al. (2021)[38]	Chai et al. (2021)[38]	Onset neonatal-infancy, axial hypotonia, spastic tetraplegia with brisk tendon reflexes, dystonia (+/-), intractable seizures	**Mouse:** Chai et al. (2021)[38]
**Pontocerebellar Hypoplasia Type 15**								
Type-15 **#**	6q21	AR	*CDC40*	Pre-mRNA splicing	Chai et al. (2021)[38]	Chai et al. (2021)[38]	Onset in infancy, developmental delays, intractable epilepsy, hypertonia, spastic tetraplegia	**Mouse**: Chai et al. (2021)[38]
**Pontocerebellar Hypoplasia Type 16**								
Type-16	10q23.2	AR	*MINPP1*	Neuroectodermal development	Ucuncu et al. (2020)[39]	Ucuncu et al. (2020)[39]	Onset in infancy, hypotonia, severe developmental delay, intractable epilepsy, spasticity, microcephaly	**Mouse**: Ucuncu et al. (2020)[39]

Supplementary Table 2 Continued. PCH Categorization.

REFERENCES

1. Barth P. Pontocerebellar hypoplasias: An overview of a group of inherited neurodegenerative disorders with fetal onset. *Brain & Development*. 1993;15(6):411-422
2. Norman RM. Cerebellar hypoplasia in Werdnig-Hoffmann disease. *Arch Dis Child*. 1961;36(185):96-101. doi:10.1136/adc.36.185.96
3. Renbaum P, Kellerman E, Jaron R, et al. Spinal muscular atrophy with pontocerebellar hypoplasia is caused by a mutation in the VRK1 gene. *Am J Hum Genet*. 2009;85(2):281-289. doi:10.1016/j.ajhg.2009.07.006
4. Vinograd-Byk H, Sapir T, Cantarero L, et al. The spinal muscular atrophy with pontocerebellar hypoplasia gene VRK1 regulates neuronal migration through an amyloid-β precursor protein-dependent mechanism. *J Neurosci*. 2015;35(3):936-942. doi:10.1523/JNEUROSCI.1998-14.2015
5. Ryan MM, Cooke-Yarborough CM, Procopis PG, Ouvrier RA. Anterior horn cell disease and olivopontocerebellar hypoplasia. *Pediatr Neurol*. Aug 2000;23(2):180-4. doi:10.1016/s0887-8994(00)00166-1
6. Wan J, Yourshaw M, Mamsa H, et al. Mutations in the RNA exosome component gene EXOSC3 cause pontocerebellar hypoplasia and spinal motor neuron degeneration. *Nat Genet*. 2012;44(6):704-708. Published 2012 Apr 29. doi:10.1038/ng.2254
7. Boczonadi V, Muller JS, Pyle A, et al. EXOSC8 mutations alter mRNA metabolism and cause hypomyelination with spinal muscular atrophy and cerebellar hypoplasia. *Nat Commun*. Jul 3 2014;5:4287. doi:10.1038/ncomms5287
8. Burns DT, Donkervoort S, Muller JS, et al. Variants in EXOSC9 Disrupt the RNA Exosome and Result in Cerebellar Atrophy with Spinal Motor Neuronopathy. *Am J Hum Genet*. May 3 2018;102(5):858-873. doi:10.1016/j.ajhg.2018.03.011
9. Wan J, Steffen J, Yourshaw M, et al. Loss of function of SLC25A46 causes lethal congenital pontocerebellar hypoplasia. *Brain*. Nov 1 2016;139(11):2877-2890. doi:10.1093/brain/aww212
10. Somashekar PH, Kaur P, Stephen J, et al. Bi-allelic missense variant, p.Ser35Leu in EXOSC1 is associated with pontocerebellar hypoplasia. *Clin Genet*. Apr 2021;99(4):594-600. doi:10.1111/cge.13928
11. Barth PG, Vrensen GF, Uylings HB, Oorthuys JW, Stam FC. Inherited syndrome of microcephaly, dyskinesia and pontocerebellar hypoplasia: a systemic atrophy with early onset. *J Neurol Sci*. Jun 1990;97(1):25-42.
12. Budde BS, Namavar Y, Barth PG, et al. tRNA splicing endonuclease mutations cause pontocerebellar hypoplasia. *Nat Genet*. Sep 2008;40(9):1113-8. doi:10.1038/ng.204
13. Namavar Y, Barth PG, Kasher PR, et al. Clinical, neuroradiological and genetic findings in pontocerebellar hypoplasia. *Brain*. Jan 2011;134(Pt 1):143-56. doi:10.1093/brain/awq287
14. Ben-Zeev B, Hoffman C, Lev D, et al. Progressive cerebellocerebral atrophy: a new syndrome with microcephaly, mental retardation, and spastic quadriplegia. *J Med Genet*. Aug 2003;40(8):e96. doi:10.1136/jmg.40.8.e96
15. Agamy O, Ben Zeev B, Lev D, et al. Mutations disrupting selenocysteine formation cause progressive cerebello-cerebral atrophy. *Am J Hum Genet*. 2010;87(4):538-544. doi:10.1016/j.ajhg.2010.09.007

16. Feinstein M, Flusser H, Lerman-Sagie T, et al. VPS53 mutations cause progressive cerebello-cerebral atrophy type 2 (PCCA2). *Journal of Medical Genetics*. 2014;51(5):303-308. doi:10.1136/jmedgenet-2013-101823
17. Breuss MW, Sultan T, James KN, et al. Autosomal-Recessive Mutations in the tRNA Splicing Endonuclease Subunit TSEN15 Cause Pontocerebellar Hypoplasia and Progressive Microcephaly. *Am J Hum Genet*. 2016;99(1):228-235. doi:10.1016/j.ajhg.2016.05.023
18. Rajab A, Mochida GH, Hill A, et al. A novel form of pontocerebellar hypoplasia maps to chromosome 7q11-21. *Neurology*. 2003;60(10):1664-1667. doi:10.1212/01.wnl.0000068548.58498.41
19. Ahmed MY, Chioza BA, Rajab A, et al. Loss of PCLO function underlies pontocerebellar hypoplasia type III. *Neurology*. Apr 28 2015;84(17):1745-50. doi:10.1212/WNL.0000000000001523
20. Falck J, Bruns C, Hoffmann-Conaway S, et al. Loss of Piccolo Function in Rats Induces Cerebellar Network Dysfunction and Pontocerebellar Hypoplasia Type 3-like Phenotypes. *J Neurosci*. 2020;40(14):2943-2959. doi:10.1523/JNEUROSCI.2316-19.2020
21. Albrecht S SM, Belmont J, Armstrong DL. Fatal infantile encephalopathy with olivopontocerebellar hypoplasia and micrencephaly. Report of three siblings.(Berl). *Acta Neuropathol* 1993;
22. Patel MS, Becker LE, Toi A, Armstrong DL, Chitayat D. Severe, fetal-onset form of olivopontocerebellar hypoplasia in three sibs: PCH type 5? *Am J Med Genet A*. Mar 15 2006;140(6):594-603. doi:10.1002/ajmg.a.31095
23. Edvardson S, Shaag A, Kolesnikova O, et al. Deleterious mutation in the mitochondrial arginyl-transfer RNA synthetase gene is associated with pontocerebellar hypoplasia. *Am J Hum Genet*. 2007;81(4):857-862. doi:10.1086/521227
24. Mahbubul Huq AH, Nigro MA. XY sex reversal and a nonprogressive neurologic disorder: a new syndrome? *Pediatr Neurol*. Oct 2000;23(4):357-60. doi:10.1016/s0887-8994(00)00200-9
25. Lardelli RM, Schaffer AE, Eggens VR, et al. Biallelic mutations in the 3' exonuclease TOE1 cause pontocerebellar hypoplasia and uncover a role in snRNA processing. *Nat Genet*. 2017;49(3):457-464. doi:10.1038/ng.3762
26. Mochida GH, Ganesh VS, de Michelena MI, et al. CHMP1A encodes an essential regulator of BMI1-INK4A in cerebellar development. *Nat Genet*. 2012;44(11):1260-1264. doi:10.1038/ng.2425
27. Akizu N, Cantagrel V, Schroth J, et al. AMPD2 regulates GTP synthesis and is mutated in a potentially treatable neurodegenerative brainstem disorder. *Cell*. Aug 1 2013;154(3):505-17. doi:10.1016/j.cell.2013.07.005
28. Karaca E, Weitzer S, Pehlivan D, et al. Human CLP1 mutations alter tRNA biogenesis, affecting both peripheral and central nervous system function. *Cell*. Apr 24 2014;157(3):636-50. doi:10.1016/j.cell.2014.02.058
29. Schaffer AE, Eggens VR, Caglayan AO, et al. CLP1 founder mutation links tRNA splicing and maturation to cerebellar development and neurodegeneration. *Cell*. Apr 24 2014;157(3):651-63. doi:10.1016/j.cell.2014.03.049
30. Hanada T, Weitzer S, Mair B, et al. CLP1 links tRNA metabolism to progressive motor-neuron loss. *Nature*. 2013;495(7442):474-480. doi:10.1038/nature11923
31. Monaghan CE, Adamson SI, Kapur M, Chuang JH, Ackerman SL. The *Clp1* R140H mutation alters tRNA metabolism and mRNA 3' processing in mouse models of pontocerebellar hypoplasia. *Proc Natl Acad Sci U S A*. 2021;118(39):e2110730118. doi:10.1073/pnas.2110730118

32. Morisaki I, Shiraishi H, Fujinami H, et al. Modeling a human CLP1 mutation in mouse identifies an accumulation of tyrosine pre-tRNA fragments causing pontocerebellar hypoplasia type 10. *Biochem Biophys Res Commun*. 2021;570:60-66. doi:10.1016/j.bbrc.2021.07.036
33. Ivanova EL, Mau-Them FT, Riazuddin S, et al. Homozygous Truncating Variants in TBC1D23 Cause Pontocerebellar Hypoplasia and Alter Cortical Development. *Am J Hum Genet*. Sep 7 2017;101(3):428-440. doi:10.1016/j.ajhg.2017.07.010
34. Marin-Valencia I, Gerondopoulos A, Zaki MS, et al. Homozygous Mutations in TBC1D23 Lead to a Non-degenerative Form of Pontocerebellar Hypoplasia. *Am J Hum Genet*. Sep 7 2017;101(3):441-450. doi:10.1016/j.ajhg.2017.07.015
35. van Dijk T, Ferdinandusse S, Ruiter JPN, et al. Biallelic loss of function variants in COASY cause prenatal onset pontocerebellar hypoplasia, microcephaly, and arthrogryposis. *Eur J Hum Genet*. Dec 2018;26(12):1752-1758. doi:10.1038/s41431-018-0233-0
36. Gershlick DC, Ishida M, Jones JR, Bellomo A, Bonifacino JS, Everman DB. A neurodevelopmental disorder caused by mutations in the VPS51 subunit of the GARP and EARP complexes. *Hum Mol Genet*. May 1 2019;28(9):1548-1560. doi:10.1093/hmg/ddy423
37. Uwineza A, Caberg JH, Hitayezu J, et al. VPS51 biallelic variants cause microcephaly with brain malformations: A confirmatory report. *Eur J Med Genet*. Aug 2019;62(8):103704. doi:10.1016/j.ejmg.2019.103704
38. Chai G, Webb A, Li C, et al. Mutations in Spliceosomal Genes PPIL1 and PRP17 Cause Neurodegenerative Pontocerebellar Hypoplasia with Microcephaly. *Neuron*. 2021;109(2):241-256.e9. doi:10.1016/j.neuron.2020.10.035
39. Ucuncu E, Rajamani K, Wilson MSC, et al. MINPP1 prevents intracellular accumulation of the chelator inositol hexakisphosphate and is mutated in Pontocerebellar Hypoplasia. *Nat Commun*. Nov 30 2020;11(1):6087. doi:10.1038/s41467-020-19919-y
